# Sugar restriction in the first 1000 days after conception, and long-term respiratory health: a quasi-experiment study

**DOI:** 10.1016/j.ajcnut.2025.09.045

**Published:** 2025-10-07

**Authors:** Jiazhen Zheng, Chuang Yang, Zhen Zhou, Jinghan Huang, Qiang Tu, Haisheng Wu, Quan Yang, Wenbo Huang, Junchun Shen, Feng Cao

**Affiliations:** 1 Bioscience and Biomedical Engineering Thrust, Systems Hub, The Hong Kong University of Science and Technology (Guangzhou), Guangzhou, Guangdong, China; 2 Department of Visceral, Transplant, Thoracic and Vascular Surgery, University Hospital Leipzig, Leipzig, Germany; 3 School of Public Health and Preventive Medicine, Monash University, Melbourne, Victoria, Australia; 4 Biomedical Genetics Section, School of Medicine, Boston University, Boston, MA, United States; 5 Department of Chemical Pathology, Faculty of Medicine, The Chinese University of Hong Kong, Ma Liu Shui, Hong Kong; 6 Faculty of Medicine and Health, The University of Sydney, Sydney, New South Wales, Australia; 7 School of Public Health, LKS Faculty of Medicine, The University of Hong Kong, Hong Kong Special Administrative Region, Hong Kong; 8 Cardiac and Vascular Center, The University of Hong Kong-Shenzhen Hospital, Shenzhen, China; 9 Department of Clinical Epidemiology & Health Economics, The University of Tokyo, Tokyo, Japan; 10 Medical Faculty, RWTH Aachen University, Aachen, Germany

**Keywords:** early-life, sugar restriction, COPD, asthma, idiopathic pulmonary fibrosis, spirometry

## Abstract

**Background:**

High sugar intake during the first 1000 d after conception is common and may impact lifelong respiratory health. Although early-life sugar exposure has been linked to childhood asthma, its long-term effect on adult respiratory disease and lung function is unclear.

**Objectives:**

This study aims to assess whether restricted sugar intake during the first 1000 d after conception is associated with risk and delayed onset of asthma, chronic obstructive pulmonary disease (COPD), and idiopathic pulmonary fibrosis (IPF) in adulthood, and to explore potential mediating mechanisms.

**Methods:**

We used the cessation of the United Kingdom’s sugar rationing in September 1953 as a quasi-experiment, comparing 58,670 UK Biobank participants born 1951–1956 exposed to varying durations of early-life sugar restriction. Participants were classified as exposed (in utero and/or ≤2 y postbirth) or unexposed (born after July 1954). Outcomes included incident asthma, COPD, and IPF (ascertained by health records), validated in the English Longitudinal Study of Ageing (a United Kingdom cohort) and the Health and Retirement Study (a United States cohort). Spirometry indices [forced expiratory volume in 1 s (FEV1)%, forced vital capacity (FVC)%, FEV1/FVC] were measured. Cox models adjusted for demographic, socioeconomic, lifestyle, and clinical factors. Mediation analysis assessed diabetes, hypertension, and birth weight.

**Results:**

Early-life exposure to sugar rationing (in utero plus 1–2 y) was associated with lower risks of asthma [hazard ratio: 0.75; 95% confidence interval (CI): 0.61, 0.91] and COPD [0.73 (0.62, 0.88)], and delayed disease onset by ≤3.6 y. Rationed participants had higher mean FEV1% (increase 6.0; 95% CI: 2.9, 9.1), FVC% [increase 5.9 (3.3, 8.5)], and FEV1/FVC ratio [increase 0.045 (0.011, 0.079)] compared with nonrationed groups. Longer sugar restriction offered greater protection. Diabetes and hypertension mediated 18.2% of the effect; birth weight contributed only 1.6%. Findings were consistent across external validation and placebo analyses.

**Conclusions:**

Restricting sugar intake during the first 1,000 d after conception was associated with reduced risk and delayed onset of asthma and COPD, and improved lung function in adulthood. These findings support the utility of current dietary guidelines to promote lifelong respiratory health.

## Introduction

The “fetal origins of respiratory disease” hypothesis is supported by an animal study linking maternal sucrose intake to adverse effects on lung growth and development in offspring [1]. Héctor Javier Nava Reyes et al. [1] found that, compared with the control group, Wistar rats receiving sucrose both before and during pregnancy showed a significant reduction in neonatal body weight and lung weight, along with changes in lung parenchyma, glycogen deposition, collagen fibers, and elastin structure. Evidence from human studies suggests that maternal sugar intake during pregnancy may be associated with childhood asthma [2]. The Avon Longitudinal Study of Parents and Children (ALSPAC) study, a prospective birth cohort of predominantly White European families in the United Kingdom, found that higher maternal sugar intake increased the odds of atopy [odds ratio (OR) 1.38] and atopic asthma (OR 2.01) in children [3]. The International Study of Asthma and Allergies in Childhood (ISAAC), a large international multicenter cross-sectional study of children from diverse ethnic backgrounds, found a linear relationship between per capita added sugar consumption and severe asthma symptoms in children aged 6–7 y [4]. Additionally, the Randomised control trial of Low glycaemic index diet in pregnancy (ROLO), observed that a low glycemic index diet during pregnancy reduced risk of asthma in children at age 5, particularly in mothers with lower educational attainment (adjusted OR 0.46) [5]. However, the long-term impacts of sugar rationing on various respiratory outcomes and spirometry results later in life remain unclear.

A previous study indicated that after the end of sugar rationing in September 1953, there was a sharp increase in the consumption of sugar and sweets. This suggests a significant surge in consumption patterns [6]. Specifically, Gracner et al. [7] found that the average daily sugar consumption for an adult markedly increased after the end of sugar rationing in September 1953, rising from 41 g during Q1 1953 to around 80 g by Q3 1954. The complete termination of rationing occurred in July 1954, whereas the intake of other foods and nutrients, except for sugar, remained stable or showed only minor changes during this period. This provided a unique opportunity to assess the long-term health impacts of restricted sugar exposure during critical developmental stages.

In this study, we examine whether limiting sugar intake during the first 1000 d is associated with lower risks and delayed onset of idiopathic pulmonary fibrosis (IPF), chronic obstructive pulmonary disease (COPD), and asthma, and whether longer periods of restricted exposure are associated with progressively greater benefit.

## Methods

### Study design and participants

**FIGURE 1:**
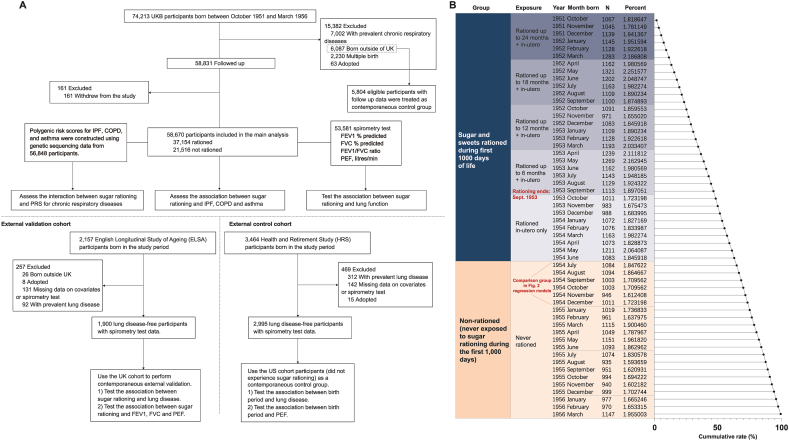
(A) Flowchart depicting the study cohort. (B) Distribution of birth samples in the UK Biobank by calendar month and their exposure to sugar rationing. The sugar-rationed group is shown in blue, whereas the group unexposed to sugar rationing is shown in orange. Actual sugar intake was not directly measured; exposure status was classified based on birth period relative to national sugar rationing policies. Specifically, individuals born between October 1951 and June 1954 were considered “rationed,” whereas those born between July 1954 and March 1956 were considered “nonrationed.” Cumulative rate (%) on the x-axis represents the cumulative proportion of the total sample, increasing from 0% at the top to 100% at the bottom as each group is sequentially added. The first group of individuals who were never exposed to sugar rationing has been labeled and used as the control group in Figure 2. COPD, chronic obstructive pulmonary disease; FEV1, forced expiratory volume in 1 s; FVC, forced vital capacity; IPF, idiopathic pulmonary fibrosis; PEF, peak expiratory flow; PRS, polygenic risk score; UKB, UK Biobank.

We used an event study approach to investigate the long-term impact of limited sugar intake during the first 1000 d after conception on chronic respiratory diseases and lung function. The year of birth determined whether an individual experienced sugar rationing during their early years. Individuals are quasi-randomly assigned to either the rationing (low sugar) group or the nonrationing (high sugar) group based on their birth year. Figure 1 illustrates the timeline of sugar rationing, dividing individuals into the rationing group and nonrationing group based on birth dates. This quasi-experimental design simulating sugar rationing has been used previously by Gracner et al. [7]. For details on the 1000-d window around the end of rationing and the regression discontinuity rationale, refer to their study [7].

We analyzed the National Food Survey (NSF) reports to examine quarterly dietary trends during and after the rationing period [[7], [8], [9]], spanning 1950–1960. Detailed annual data on food intake, Food Price Index, Consumer Price Index, and related socioeconomic indicators are available in Supplemental Material 2 or via https://www.gov.uk/government/statistics/family-food-historic-reports.

The objectives, participant characteristics, and data collection procedures of the UK Biobank (UKB) have been described in previous publications [10]. Briefly, between 2006 and 2010, the UKB enrolled >500,000 individuals aged 40–70 from the general population across 22 assessment centers in the United Kingdom. Data collection involved surveys, interviews, periodic visits to assessment centers, and linkage to health records, capturing a wide range of physiological, psychological, social, and health-related information [10]. The study received ethical approval from the Northwest Multi-Center Research Ethics Committee, and all participants provided informed consent at the first visit. The UKB application number for this study is 97089.

A total of 74,213 UKB participants born between October 1951 and March 1956 were initially considered for inclusion. After excluding 15,382 individuals (7002 with prevalent chronic respiratory diseases, 6087 born outside the United Kingdom, 2230 from multiple births, and 63 adopted), and a further 161 who withdrew from the study, 58,670 participants were included in the main analysis (Figure 1).

### Early-life exposure to sugar rationing and covariates

The exposure to rationing was categorized based on its duration into the following groups: no exposure, exposure only in utero, and exposure in utero and ≤6, 12, 18, or 24 mo after birth. To improve statistical robustness, broader categories were also created: exposure limited to in utero, exposure in utero and the first year after birth, exposure in utero and 1–2 y after birth, or no exposure. The timeline of rationing exposure and the distribution of births across the entire study period are shown in Figure 1.

All participants in the UKB cohort (including both exposure and control groups) visited 1 of the 22 assessment centers in the United Kingdom, where they completed an interactive questionnaire collecting information on sex, age, race, place of birth (England, Wales, Scotland, or outside the United Kingdom), household income, education, Townsend deprivation index, smoking habits, alcohol consumption, physical activity, parental health history, and early-life conditions. Place of birth within the United Kingdom (recorded as North and East coordinates) and birth weight were collected through interviews conducted by trained personnel. Cardiovascular diseases, digestive diseases, kidney diseases, and liver diseases were diagnosed using the International Classification of Diseases, 10th edition (ICD-10).

Below is detailed information regarding the covariates: The BMI is determined by dividing a person's weight (in kilograms) by the square of their height (in meters), resulting in a unit of kg/m^2^. The Townsend deprivation index is an indicator of socioeconomic hardship, [11] based on factors such as employment status, car ownership, housing conditions, and household overcrowding. Hypertension was defined as: *1*) antihypertensive medication use, *2*) systolic BP > 140 mmHg, *3*) diastolic BP > 90 mmHg, or *4*) self-reported diagnosis. High cholesterol required either self-reported diagnosis, lipid-lowering medication use, or total cholesterol ≥200 mg/dL. Diabetes was classified by self-reported diagnosis, antidiabetic medication use, glycated hemoglobin (HbA1c) ≥6.5%, or fasting glucose ≥126 mg/dL. For further information on the calculation of polygenic risk score (PRS) and East and North coordinates, refer to the Supplemental Methods. Additional details about these measurements are available on the UKB website (www.ukbiobank.ac.uk).

### Evaluation of chronic respiratory outcomes and placebo outcomes

In this study, the primary outcomes include IPF, COPD, and asthma. Dates of death and causes were obtained by linking to the National Health Service (NHS) death registers in England and Wales, as well as the NHS central registry in Scotland. Additionally, hospitalization dates and reasons were determined through linkage to Scottish morbidity records for Scottish participants and hospital morbidity statistics for participants in England and Wales [10]. Respiratory diseases were defined using the ICD-10 codes: IPF was defined by code J84.1, COPD by code J44, and asthma by code J45.

Our placebo outcomes focused on diseases that were less likely to be influenced by early dietary factors, including influenza (defined by codes J09–J11) and herpes zoster (defined by codes B02). The follow-up period for all participants began at recruitment (March 2006 and July 2010) and ended at the time of outcome diagnosis, death, loss to follow-up, or study completion (1 July, 2023), whichever occurred first. Health and Retirement Study (HRS) and English Longitudinal Study of Ageing (ELSA) identified lung diseases through biennial surveys, where participants answered questions posed by the interviewer regarding whether a doctor had ever told them they had a lung disease. Apart from differences in birth period, the methods for data collection, survey administration, and outcome ascertainment were identical across all exposure and control groups.

### Spirometry testing

In the UKB study, all participants were required to undergo spirometry testing at recruitment. Participants were instructed to perform 2 to 3 blows using a pneumotachometer (Pneumotrac 6800; Vitalograph) under the supervision of trained technicians. The first 2 blows were compared for reproducibility, and if the difference was within an acceptable range (defined as <5%), the third blow was skipped. The researchers assessed the spirometry results, using the highest forced expiratory volume in 1 s (FEV1), forced vital capacity (FVC), and peak expiratory flow values from the acceptable blows (the best measurement). We utilized the RSpiro package in R version 4.0.2 (R Foundation for Statistical Computing) to calculate the predicted FEV1% and FVC% values, [12] based on the Global Lung Initiative 2012 reference values. Further information on spirometry measurements in the HRS and ELSA cohorts is provided in the Supplemental Methods.

### External validation and control cohorts

A frequent issue in such studies is the possibility of unaccounted differences between groups both during and after the sugar rationing period. To address this issue, we designated an internal contemporaneous control group within the UKB: 5804 eligible participants born outside the United Kingdom with available follow-up data (2262 born July 1954–March 1956; 3542 born October 1951–June 1954). In addition, we utilized 2 external longitudinal datasets, the HRS [13] as a control dataset and the ELSA [14] as an external validation. These datasets represent the aging populations of the United States and the United Kingdom, respectively. Participants are interviewed every 2 y using standardized questionnaires and similar measurement tools to assess economic, physical, and mental health. We collected data from waves 4–12 of HRS (1998–2014) and waves 1–9 of ELSA (2002–2018). The fourth wave of HRS (1998) and the first wave of ELSA (2002) were considered baseline data. Given that the design objectives of these surveys were harmonized, they provide an opportunity for cross-national research. More details about the HRS and ELSA cohorts can be found in the Supplemental Methods. The inclusion and exclusion criteria are shown in Figure 1.

Our main hypothesis is that exposure to sugar rationing during the first 1000 d after conception is associated with a lower risk and later onset of lung diseases, as well as better adult lung function. Our subsidiary hypothesis for the control populations is that differences in birth periods within these controls are not associated with respiratory diseases or lung function. The validation group, born during the same period as the study population in the United Kingdom, serves to test whether differing birth periods in this population have a significant effect on respiratory health, at the very least, exhibit similar trends to those in the study population.

### Statistical analysis

Detailed information on statistical analysis is provided in the Supplemental Methods. To summarize the data, continuous variables are presented as means ± SD, whereas categorical variables are reported as frequencies (percentages) (Table 1). Missing values are shown in Supplemental Table 1.

**TABLE 1:** Baseline characteristics of participants born between October 1951 and March 1956.

	Total	Not rationed	Rationed
No. of participants	58,670	21,516	37,154
Age at entry (y), mean (SD)	54.6 (1.6)	53.2 (1.0)	55.4 (1.2)
Females	33,060 (56.3)	12,160 (56.5)	20,900 (56.3)
Place of birth			
England	50,448 (86.0)	18,432 (85.7)	32,016 (86.2)
Scotland	5347 (9.1)	2059 (9.6)	3288 (8.8)
Wales	2875 (4.9)	1025 (4.8)	1850 (5.0)
Birth month			
1 March–31 May	15,376 (26.2)	4462 (20.7)	10,914 (29.4)
1 June–31 August	13,271 (22.6)	5280 (24.5)	7991 (21.5)
1 September–30 November	14,218 (24.2)	5837 (27.1)	8381 (22.6)
1 December–28 February	15,805 (26.9)	5937 (27.6)	9868 (26.6)
White (%)	56,432 (96.2)	20,640 (95.9)	35,792 (96.3)
Education (%)			
Below A levels	24,339 (41.5)	8775 (40.8)	15,564 (41.9)
A levels	18,299 (31.2)	6984 (32.5)	11,315 (30.5)
College/university	7182 (12.2)	2629 (12.2)	4553 (12.3)
Professional/other	8850 (15.1)	3128 (14.5)	5722 (15.4)
Household income (£)			
<18,000	13,926 (23.7)	4759 (22.1)	9167 (24.7)
18,000–30,999	10,984 (18.7)	3793 (17.6)	7191 (19.4)
31,000–51,999	15,500 (26.4)	5743 (26.7)	9757 (26.3)
52,000–100,000	14,168 (24.1)	5575 (25.9)	8593 (23.1)
>100,000	4092 (7.0)	1646 (7.7)	2446 (6.6)
Townsend deprivation index, mean (SD)	−1.4 (3.0)	−1.4 (3.0)	−1.5 (3.0)
Smoking status (%)			
Current	6552 (11.2)	2577 (12.0)	3975 (10.7)
Never	32,844 (56.0)	12,202 (56.7)	20,642 (55.6)
Previous	19,274 (32.9)	6737 (31.3)	12,537 (33.7)
Pack-years of smoking, mean (SD)	22.6 (17.6)	22.1 (16.9)	22.9 (18.0)
Consumption of alcohol (%)			
Current	54,222 (92.4)	19,966 (92.8)	34,256 (92.2)
Never	2457 (4.2)	860 (4.0)	1597 (4.3)
Previous	1991 (3.4)	690 (3.2)	1301 (3.5)
BMI (kg/m^2^), mean (SD)	27.5 (5.0)	27.5 (5.0)	27.5 (5.0)
Summed MET min/wk for all activity, mean (SD)	2501.7 (2410.6)	2506.7 (2451.9)	2498.7 (2386.4)
Personal medical condition			
Cardiovascular disease	2169 (3.7)	680 (3.2)	1489 (4.0)
Hypertension	32,620 (55.6)	11,646 (54.1)	20,974 (56.5)
High cholesterol	10,269 (17.5)	3541 (16.5)	6728 (18.1)
Diabetes	2973 (5.1)	1037 (4.8)	1936 (5.2)
Digestive disease	512 (0.9)	174 (0.8)	338 (0.9)
Kidney disease	1578 (2.7)	529 (2.5)	1049 (2.8)
Liver disease	2074 (3.5)	725 (3.4)	1349 (3.6)
Parents' condition			
Parents diagnosed with lung disease	14,905 (25.4)	5224 (24.3)	9681 (26.1)
Parents still alive	9246 (15.8)	4311 (20.0)	4935 (13.3)
Maternal smoking around birth	18,019 (30.7)	6608 (30.7)	11,411 (30.7)
Breastfed as an infant	34,260 (58.4)	12,472 (58.0)	21,788 (58.6)
Birth weight (kg), mean (SD)	3.3 (0.5)	3.3 (0.5)	3.3 (0.5)

Data are presented as no. (%) or mean (SD).

Abbreviation: MET, metabolic equivalent of task.

Model 1 to model 3 were Cox proportional hazard model.

In model 1, we adjusted for age and sex.

In model 2, we included DAG-selected covariates, including age, sex, race, birth location, calendar month of birth, household income, Townsend deprivation index, real food prices (adjusted for the consumer price index), parental lung disease, whether parents still alive, maternal smoking around birth, whether breastfed as an infant and the social average fat intake around the period of birth (derived from the NSF).

In model 3, we further adjusted baseline lifestyle factors (smoking status, alcohol intake, and physical activity), baseline medical conditions (CVD, hypertension, diabetes, digestive disease, kidney disease, and liver disease), and survey year.

Model 4 adjusted for terms in model 3 and used parametric hazard models based on the Gompertz distribution.

Abbreviations: CI, confidence interval; COPD, chronic obstructive pulmonary disease; CVD, cardiovascular disease; DAG, directed acyclic graph; HR, hazard ratio; IPF, idiopathic pulmonary fibrosis; NSF, National Food Survey.

To minimize bias in inference, we applied multiple imputation using chained equations across 20 datasets, [15] with the process outlined in the Supplemental Methods. The chi-square test was used to determine *P* values for categorical variables, whereas the Mann–Whitney U test was employed for continuous variables to assess differences between rationed and nonrationed groups.

To estimate associations between sugar rationing and incident chronic respiratory diseases, we fit Cox proportional hazards models and parametric survival models assuming a Gompertz distribution, reporting hazard ratios (HRs) with 95% confidence intervals (CIs). The proportional hazards assumption was evaluated using Schoenfeld residual plots, and no deviations from the assumption were observed [16]. After assessing the best-fit distribution using Akaike information criterion and Bayesian information criterion, the Gompertz distribution was selected (Supplemental Table 2) [17].

To guide covariate adjustment, we used a directed acyclic graph (DAG) (Supplemental Figure 1). Model 1 adjusted for age and sex. Model 2 further included DAG-selected covariates: demographic factors (age, sex, race, birth location, birth month), socioeconomic status (household income, Townsend deprivation index), inflation-adjusted food prices, parental lung disease and survival status, early-life exposures (maternal smoking, breastfeeding), and social average fat intake during birth year (from NSF). Model 3 additionally adjusted for baseline lifestyle factors (smoking, alcohol, physical activity), medical conditions [cardiovascular disease (CVD), hypertension, diabetes, digestive/kidney/liver diseases] and survey year, aiming to better isolate sugar rationing's effect on lung health.

To assess robustness, we conducted 3 sensitivity analyses: *1*) we further adjusted for annual mean black smoke/particulate matter with diameter < 10 μm (PM_10_) concentrations, using historical data measured in London as reported by Lam et al. [18] *2*) We also re-estimated the associations using Cox models that included only birth and early-life covariates, excluding all mid- or later-life variables, to further minimize potential bias from endogenous variables. *3*) We performed logistic regression including all individuals, regardless of baseline chronic respiratory disease status, to examine whether our findings were robust to survivor and selection bias.

To account for potential biases arising from general time trends or improvements in disease detection, we compared the outcomes of adults born at 6-mo intervals after the end of sugar rationing in the primary analysis. Our main hypothesis was that, after controlling for covariates, the disease risk for adults born after December 1954, who were not exposed to sugar rationing, would be similar, with an HR close to 1.

To address effect heterogeneity, in the subgroup analysis, we assessed potential modification effects based on several factors, including sex (male, female), race (White, other groups), place of birth (England, Wales, Scotland), and the PRS for IPF, COPD, or asthma (above or below the median). Additionally, we considered whether participants' parents had been diagnosed with lung disease, whether their mothers smoked before or after birth (yes, no), and whether they were breastfed as infants (yes, no). We stratified participants by deciles of east and north birth coordinates to explore potential spatial variation in the association between early-life sugar rationing exposure and respiratory disease risk. Heterogeneity across spatial deciles was assessed using random-effects meta-analysis with calculation of *I*^2^, *τ*^2^, and *Q*-test *P* values.

To examine mediation, we conducted structural equation modeling with the lavaan R package, [19] to investigate the proportion mediated by type 2 diabetes, hypertension, and birth weight in the relationship between sugar rationing and lung disease. The detailed steps are shown in the Supplemental Methods.

**FIGURE 2:**
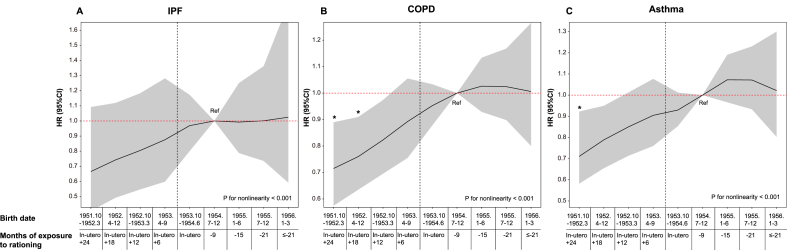
Hazard ratios (HRs) for various chronic respiratory diseases across different levels of exposure to rationing. Parametric hazard models based on the Gompertz distribution were applied. Each point represents a HR comparing the hazard rate of adults exposed to rationing in utero, in utero plus 6/12/18/24 mo after birth, or not exposed to rationing, with the reference group of adults born between July and December 1954. The group never exposed to rationing includes individuals born between January and June 1955 (≥ 15 mo after rationing), July and December 1955 (≥ 21 mo after rationing), and January and March 1956. HR estimates for adults born between January 1955 and April 1956, who were never exposed to rationing, were not significantly different from those of the reference group (*P* = 0.282, 0.361, and 0.310 for IPF, COPD, and asthma, respectively). We adjusted for age, sex, race, birth location, calendar month of birth, household income, Townsend deprivation index, real food prices (adjusted for the consumer price index), parental lung disease, whether parents still alive, maternal smoking around birth, whether breastfed as an infant, the social average fat intake around the period of birth (derived from the NSF), baseline lifestyle factors (smoking status, alcohol intake and physical activity), baseline medical conditions (CVD, hypertension, diabetes, digestive disease, kidney disease, and liver disease) and survey year. The shaded area represents the 95% confidence interval. The vertical dashed black line marks the end of sugar rationing. ∗Statistically significant after Bonferroni correction for multiplicity (*P* < 0.0167). CI, confidence interval; COPD, chronic obstructive pulmonary disease; CVD, cardiovascular disease; IPF, idiopathic pulmonary fibrosis; NSF, National Food Survey.

**FIGURE 3:**
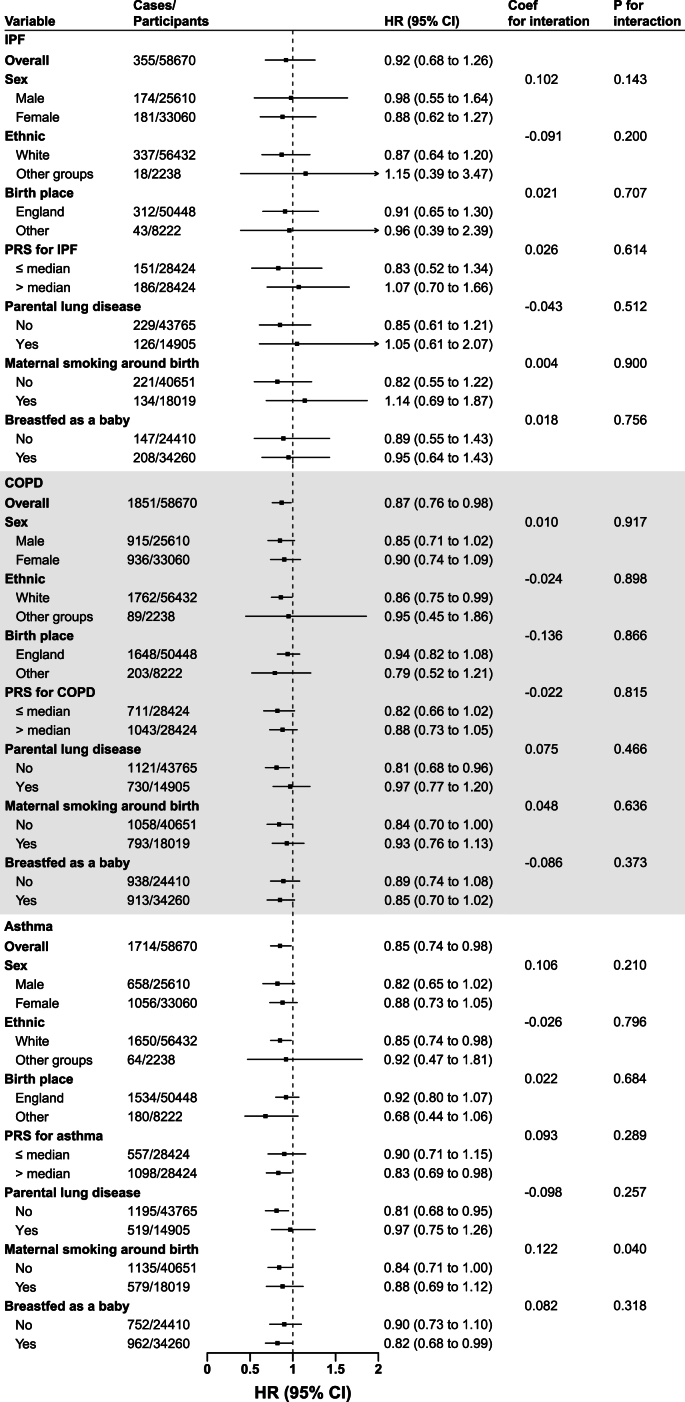
Stratified analysis examining the association between sugar rationing and the risk of various chronic respiratory diseases. Parametric hazard models based on the Gompertz distribution were utilized. We adjusted for age, sex, race, birth location, calendar month of birth, household income, Townsend deprivation index, real food prices (adjusted for the consumer price index), parental lung disease, whether parents still alive, maternal smoking around birth, whether breastfed as an infant, the social average fat intake around the period of birth (derived from the NSF), baseline lifestyle factors (smoking status, alcohol intake and physical activity), baseline medical conditions (CVD, hypertension, diabetes, digestive disease, kidney disease, and liver disease), and survey year. We applied a Bonferroni correction to account for multiple comparisons (21 tests), setting the significance threshold at *P* < 0.0024 (0.05/21). ∗PRS analysis was conducted in 56,848 participants with genetic sequencing data. CI, confidence interval; COPD, chronic obstructive pulmonary disease; CVD, cardiovascular disease; HR, hazard ratio; IPF, idiopathic pulmonary fibrosis; NSF, National Food Survey; PRS, polygenic risk score.

To account for potential overall trends or spurious associations that could influence our results, we re-estimated the full models for the placebo outcomes of influenza and herpes zoster. Ordinary least squares regression was used to examine the relationship between sugar rationing and spirometry measurements, controlling for the covariates listed above. We applied the Fine and Gray model to adjust for competing risks, using nonrespiratory disease mortality as a competing event [20]. Additionally, we assumed a Gompertz distribution for the event-time model to estimate the impact of sugar rationing on the delayed onset age of disease [17]. All statistical analyses were performed using R version 4.0.2 (R Core Team), with a significance level set at *P* < 0.05 (2-tailed). We evaluated 3 coprimary outcomes, IPF, COPD, and asthma, using Cox proportional hazards models. To control the family-wise type I error across these 3 outcomes, we applied a Bonferroni correction (α family = 0.05), yielding a per-outcome threshold of *P* < 0.0167 (reported in Table 2 and Figure 2). In subgroup analysis (Figure 3), for heterogeneity, we prespecified interaction tests between exposure and 7 baseline factors (sex, ethnicity, birthplace, disease-specific PRS, parental lung disease, maternal smoking around birth, and breastfeeding in infancy). Because 21 interaction tests were performed (7 factors × 3 outcomes), we used a Bonferroni-corrected threshold of *P* < 0.0024 (0.05/21).

**Table 2:** Associations of sugar rationing on risk of various chronic respiratory diseases.

	Not Rationed	In utero	In utero + (0, 1] year	In utero + (1, 2] year	P for trend
IPF					
Total sample size	21516	9660	13630	13864	
Number of cases	120	62	87	86	
Person-years	299015	133919	188680	191308	
Model 1 HR (95%CI)	Reference	1.00 (0.71-1.39)	0.89 (0.62-1.29)	0.78 (0.50-1.21)	0.254
Model 2 HR (95%CI)	Reference	0.98 (0.70-1.37)	0.87 (0.61-1.25)	0.75 (0.48-1.16)	0.183
Model 3 HR (95%CI)	Reference	1.00 (0.71-1.40)	0.90 (0.62-1.30)	0.77 (0.49-1.21)	0.245
Model 4 HR (95%CI)	Reference	0.98 (0.70-1.37)	0.86 (0.59-1.23)	0.72 (0.46-1.13)	0.145
COPD					
Total sample size	21516	9660	13630	13864	
Number of cases	685	317	438	411	
Person-years	296336	132582	186804	188894	
Model 1 HR (95%CI)	Reference	0.94 (0.81-1.08)	0.85 (0.73-1.00)	0.73 (0.60-0.89) ∗	0.002
Model 2 HR (95%CI)	Reference	0.90 (0.78-1.05)	0.81 (0.70-0.95)	0.68 (0.56-0.82) ∗	0.000
Model 3 HR (95%CI)	Reference	0.97 (0.83-1.12)	0.89 (0.76-1.05)	0.75 (0.62-0.91) ∗	0.005
Model 4 HR (95%CI)	Reference	0.92 (0.79-1.06)	0.83 (0.71-0.97)	0.73 (0.62-0.88) ∗	0.001
Asthma					
Total sample size	21516	9660	13630	13864	
Number of cases	632	273	409	400	
Person-years	294432	131975	186084	188689	
Model 1 HR (95%CI)	Reference	0.86 (0.74-1.01)	0.85 (0.72-1.00)	0.75 (0.61-0.91) ∗	0.007
Model 2 HR (95%CI)	Reference	0.86 (0.73-1.00)	0.84 (0.71-0.99)	0.73 (0.60-0.89) ∗	0.003
Model 3 HR (95%CI)	Reference	0.89 (0.76-1.04)	0.90 (0.77-1.06)	0.82 (0.67-1.01)	0.080
Model 4 HR (95%CI)	Reference	0.86 (0.74-1.00)	0.85 (0.72-1.00)	0.75 (0.61-0.91) ∗	0.006

Model 1 to Model 3 were cox proportional hazard model. In model 1, we adjusted for age and sex. In model 2, we included DAG-selected covariates, including age, sex, race, birth location, calendar month of birth, household income, Townsend deprivation index, real food prices (adjusted for the consumer price index), parental lung disease, whether parents still alive, maternal smoking around birth, whether breastfed as a baby and the social average fat intake around the period of birth (derived from the NSF). In model 3, we further adjusted baseline lifestyle factors (smoking status, alcohol intake and physical activity), baseline medical conditions (CVD, hypertension, diabetes, digestive disease, kidney disease, and liver disease) and survey year. Model 4 adjusted for terms in Model 3 and used parametric hazard models based on the Gompertz distribution. P-values less than 0.0167 (after Bonferroni correction for three primary outcomes) are indicated with an asterisk (∗). IPF = idiopathic pulmonary fibrosis; COPD = chronic obstructive pulmonary disease.

## Results

### Postrationing dietary shifts in the United Kingdom

This part does not present a novel finding; rather, the postrationing trend of increased sugar intake in the United Kingdom has already been reported by Gracner et al. [7]. We have recollected and visualized these dietary shifts primarily for clarity and ease of interpretation. According to NSF data, sugar intake rose by over 50% postrationing, whereas fat consumption showed a modest increase, and most other commodities changed minimally (Supplemental Figure 2A). After sugar rationing ended, the total calorie increase was relatively small (∼ 5% overall; see Supplemental Figure 2B). The total calorie changes stemmed chiefly from sugar intake, with a lesser contribution from fat (Supplemental Figure 2C). Supplemental Figure 2D showed that sugar consumption patterns across the 4 social classes (A, B, C, D) remained remarkably consistent.

### Participant characteristics

The study included 58,670 participants, with 21,516 in the nonrationed group and 37,154 in the rationed group (Table 1). The rationed group was slightly older (55.4 compared with 53.2 y) and had a higher proportion born between March and May (29.4% compared with 20.7%). The rationed group also had lower household incomes (proportion of those earning <£18,000: 24.7% compared with 22.1%) and a lower Townsend deprivation index (−1.5 compared with −1.4), fewer current smokers (10.7% compared with 12.0%), and a higher prevalence of comorbidities, as well as a higher proportion of parents diagnosed with lung disease (26.1% compared with 24.3%). There were no significant differences in maternal smoking around birth, birth weight, or maternal smoking during birth (*P* = 0.997, 0.111, 0.688).

### Association between early-life sugar rationing exposure and respiratory disease

In fully adjusted models (model 4) and using adults not exposed to sugar rationing as the reference group, the HRs were as follows. For IPF, the HRs were 0.98 (95% CI: 0.70, 1.37) for exposure in utero, 0.86 (0.59, 1.23) for in utero + 0–1 y, and 0.72 (0.46, 1.13) for in utero + 1–2 y (*P* for trend = 0.145) (Table 2). For COPD, the HR decreased from 0.92 (0.79, 1.06) for exposure solely in utero to 0.73 (0.62, 0.88) for in utero + 1–2 y (*P* for trend = 0.001). For asthma, a similar pattern was observed, with HRs of 0.86 (0.74, 1.00) for exposure solely in utero and 0.75 (0.61, 0.91) for in utero + 1–2 y (*P* for trend = 0.006). Across outcomes, the HRs for the in-utero-only group did not exclude the null.

For all 3 diseases, from in utero plus 24 mo to in utero only, HR for sugar rationing remained below 1 (Figure 2). Because the duration of exposure decreased, the association with lower disease risk gradually attenuated, and HRs approached 1 before stabilizing during the nonrationing period (*P* for nonlinearity < 0.05 for all outcomes). In contrast, for the nonrationed group, when comparing the incidence of disease at several time points, no significant differences were found for IPF, COPD, or asthma, with *P* values of 0.282, 0.361, and 0.310, respectively.

The density distributions of the PRS for IPF, COPD, and asthma were similar between the rationed and nonrationed groups (Supplemental Figure 3). The association between sugar rationing exposure and respiratory disease risk was not significantly influenced by sex, ethnicity, place of birth, PRS, parental lung disease, maternal smoking around birth, or breastfeeding as an infant for IPF and COPD (all *P* for interaction > 0.05) (Figure 3). For asthma, the association appeared numerically stronger in individuals without maternal smoking around birth, but the *P* value for interaction (*P* = 0.04) did not reach statistical significance after Bonferroni correction for multiple comparisons. HRs across North and East coordinate deciles showed some variation, but meta-analysis revealed no statistically significant heterogeneity (*I*^2^ = 0%, *τ*^2^ = 0, *Q*-test *P* > 0.05 for all outcomes) (Supplemental Figure 4).

Supplemental Table 3 shows the association between sugar rationing exposure and the age at onset of disease. For all 3 diseases, progressively longer delays in onset were observed with longer exposure to sugar rationing. Specifically, for participants exposed to sugar rationing in utero plus 1–2 y after birth, the onset of IPF, COPD, and asthma was delayed by 2.74, 2.85, and 3.62 y, respectively, compared with the unexposed group.

### Associations between rationing exposure and spirometry indices

Model 3 results show that sugar rationing was associated with significantly improved lung function: FEV1% predicted increased by 6.03 (95% CI: 2.87, 9.13), FVC% predicted by 5.88 (95% CI: 3.30, 8.46), and the FEV1/FVC ratio by 0.045 (95% CI: 0.011, 0.079), with all associations remaining statistically significant (*P* < 0.05) (Table 3). Overall, the density curves largely overlap, indicating only minor differences in the distribution of these spirometry indices based on sugar rationing exposure (Supplemental Figure 5).

**TABLE 3:** Associations between sugar rationing and lung function indices at UK Biobank spirometry study (*n* = 53,581).

Spirometry	Not rationed^1^	Rationed^1^	Rationed vs. notrationed-coefficient(95% CI) for model 1	Rationed vs notrationed-coefficient(95% CI) for model 2	Rationed vs notrationed-coefficient(95% CI) for model 3
FEV1 % predicted	92.81 ± 21.36	98.87 ± 21.78	6.13 (2.95, 9.26)	6.21 (3.01, 9.42)	6.03 (2.87, 9.13)
FVC % predicted	97.88 ± 22.71	103.83 ± 23.32	5.91 (3.36, 8.41)	5.97 (3.35, 8.52)	5.88 (3.30, 8.46)
FEV1/FVC ratio	0.72 ± 0.07	0.77 ± 0.07	0.048 (0.013, 0.083)	0.043 (0.008, 0.077)	0.045 (0.011, 0.079)
PEF (L/min)	392.35 ± 130.95	401.99 ± 130.29	9.15 (−0.79, 19.08)	9.02 (−0.83, 18.89)	9.20 (−0.66, 19.03)

We estimated the link between rationing exposure and lung function measurements using ordinary least squares.

In model 1, we adjusted for age and sex.

In model 2, we included DAG-selected covariates, including age, sex, race, birth location, calendar month of birth, household income, Townsend deprivation index, real food prices (adjusted for the consumer price index), parental lung disease, whether parents still alive, maternal smoking around birth, whether breastfed as an infant, and the social average fat intake around the period of birth (derived from the NSF).

In model 3, we further adjusted baseline lifestyle factors (smoking status, alcohol intake, and physical activity), baseline medical conditions (CVD, hypertension, diabetes, digestive disease, kidney disease, and liver disease), and survey year.

Abbreviations: CI, confidence interval; DAG, directed acyclic graph; FEV1, forced expiratory volume in 1 s; FVC, forced vital capacity; NSF, National Food Survey; PEF, peak expiratory flow.

1 Values are displayed as mean ± SD.

### Sensitivity analysis, placebo tests, and mediation analysis

There were 3164 competing risk events (nonrespiratory mortality) recorded for IPF, 2879 for COPD, and 3166 for asthma. The results of competing risk models closely resembled our main analysis (Supplemental Figure 6). For COPD and asthma, individuals exposed to sugar rationing consistently had lower subdistribution hazard ratios (SHRs) compared with nonrationed group, with SHRs of 0.87 (95% CI: 0.75, 1.00) for COPD and 0.86 (95% CI: 0.75, 0.98) for asthma. Further adjustment for annual black smoke/PM_10_ concentrations slightly attenuated the associations, but did not materially alter our main findings (Supplemental Table 4). Analyses restricted to birth and early-life covariates, or including individuals with prevalent chronic respiratory diseases at baseline, yielded results consistent with the main findings (Supplemental Tables 5 and 6).

The HRs for the 2 placebo outcomes, influenza and herpes zoster, are shown across various durations of rationing exposure. Throughout all exposure periods, the HR estimates remain close to 1, with their broad CIs consistently including 1 (Supplemental Figure 7).

In Supplemental Figure 8, we found that incident type 2 diabetes and incident hypertension together explained 14.5% and 10.4% of the association between sugar rationing exposure and lung disease risk, respectively. When these mediators were incorporated jointly, they accounted for 18.2% of the association, whereas birth weight contributed only 1.6%. The structural equation models used in mediation analyses showed good fit to the data, as indicated by root mean square error of approximation values below 0.05, Comparative fit index and Tucker-Lewis index values above 0.95, and standardized root mean square residual values below 0.05 for all models (Supplemental Table 7).

### Contemporaneous validation and control cohorts

The baseline characteristics of the UKB (internal control), HRS (external control), and ELSA (external validation) cohorts are presented in Supplemental Tables 8–10. In both the UKB control cohort and HRS, birth period had no significant association with lung disease incidence, with HR of 0.97, 1.04, and 1.06 for IPF, COPD, and asthma (UKB) (Supplemental Figure 9, Supplemental Table 11), and 1.06 for lung disease (HRS) (Supplemental Table 12). In ELSA, we also did not observe a statistically significant association between sugar rationing exposure and lung disease risk (HR: 0.86; 95% CI: 0.56, 1.32; Supplemental Table 13).

In the UKB control cohort and HRS, there were no significant differences in spirometry indices between the rationed and nonrationed groups (*P* > 0.05) (Supplemental Tables 14 and 15). In ELSA, the FVC coefficient for the rationed compared with nonrationed groups was 0.131 (95% CI: 0.015, 0.246, *P* = 0.026) (Supplemental Table 16), which supports the findings from the main analysis.

## Discussion

In this quasi-experimental study, we found that exposure to limited sugar intake during the first 1000 d after conception was associated with progressively lower risks of COPD and asthma as the duration of exposure increased. Additionally, the association between early-life exposure and outcomes was further evidenced by a later onset of disease. The observed association of limited sugar intake with lower risk appeared independent of genetic predisposition to these diseases. The study’s contemporaneous control cohorts, including the UKB internal control, HRS, and ELSA, consistently supported the specificity of the findings. In contrast, our placebo outcomes, including influenza and herpes zoster, showed no significant associations, reinforcing the specificity of the observed patterns. Spirometry tests revealed that sugar rationing was associated with better lung indices.

Our study found that limited sugar intake during the first 1000 d after conception protected against asthma in adulthood, with an HR of 0.75. The ALSPAC study found that the odds ratios for the highest versus lowest quintile of maternal sugar intake were 1.38 for atopy and 2.01 for atopic asthma in children [3]. The ISAAC study found a logarithmic linear relationship between severe asthma symptoms (%) in children aged 6–7 y and per capita added sugar consumption (kg/person/y) (coefficient 1.020) [4]. Furthermore, the ROLO trial found that a low glycemic index diet during pregnancy in mothers who did not complete tertiary level education was associated with a reduced risk of asthma in their children at age 5 (adjusted OR 0.46) [5]. The parallel findings from these 3 studies, together with our results, underscore the importance of early-life dietary factors in shaping the risk of respiratory conditions. Our study extends this understanding to include COPD, highlighting that early-life sugar control was associated with a lower risk across a broader range of respiratory diseases. However, the associations observed for in utero exposure alone were not statistically significant, suggesting that a longer duration of early-life sugar rationing, extending into infancy, may be required to detect measurable associations.

Our findings indicate a potential association between early-life sugar restriction and improved long-term lung function. These results align with the findings of Yang et al. [21] who observed that maternal gestational diabetes mellitus was inversely associated with FEV1 (*β* = −0.27) and FVC (*β* = −0.32) in children without respiratory diseases. Although the study by Yang et al. [21] was conducted in an Asian cohort, the similar trends observed across both studies suggest the overall benefits of early-life sugar control for lung function across different ethnic groups. Furthermore, although our study assessed population-level reductions in sugar intake during both pregnancy and infancy, the prospective cohort by Yang et al. [21] focused on maternal hyperglycemia during mid-gestation and demonstrated a reduction in offspring lung function at age 6, particularly in girls. These findings are consistent in showing that excessive maternal glucose exposure, whether from overall dietary intake or impaired glycemic control, can adversely affect lung growth and capacity in the offspring.

During the rationing period, sugar allowances for all individuals, including pregnant females and children, were notably consistent with modern dietary recommendations. Adult sugar intake was limited to under 40 g/d, closely aligning with the WHO guideline to keep free sugars below 10% of total daily energy intake (∼50 g for a 2000 kcal diet) [22]. Importantly, during this period, no added sugars were permitted for infants under 2 y old, a restriction that mirrors updated guidelines emphasizing the importance of minimizing sugar intake for infants <2 y old [23]. By inadvertently mirroring these contemporary nutritional principles, our findings transcend the historical context of sugar rationing and reveal the lung impact from present-day limits advocated by the WHO, the United States dietary guidelines, and the American Heart Association [[22], [23], [24]]. Furthermore, these findings support recent policy initiatives such as sugar taxation and stricter regulations on sugar content in maternal and infant foods to reduce chronic disease risk from early life onward [25]. Implementation of such measures, in line with WHO recommendations, may contribute to the long-term prevention of respiratory diseases at the population level [26].

### Strengths and limitations of this study

#### Strengths

Our study has several notable strengths. First, by leveraging a quasi-experimental design based on historical sugar rationing policy, we were able to examine the long-term associations between early-life sugar exposure and respiratory health, with clear temporal ordering between exposure and outcome. Second, we incorporated comprehensive data on socioeconomic status, lifestyle, parental health, and genetics, and evaluated the robustness of our findings across a range of analytical models and specifications. Third, the large sample size enabled us to examine exposure effects across different critical developmental windows. Fourth, the combination of clinical outcomes and spirometry provided insights into both subclinical and clinical endpoints. Finally, the use of placebo outcomes contributed to the specificity of our findings.

#### Limitations

This study has several important limitations. First, the underlying biological mechanisms linking early-life sugar exposure to adult respiratory outcomes remain unclear and require further research. Second, although we adjusted for many potential confounders, unmeasured confounding or secular trends, including improvements in disease detection and the influence of other food rationing, cannot be fully excluded. Third, our analysis is subject to right censoring, and we lack data on prestudy mortality; as a result, individuals who died before recruitment are not captured, which may introduce survivor bias. Fourth, unobserved differences between rationed and nonrationed groups during and after the rationing period may have introduced bias that contemporaneous control groups cannot completely eliminate. Fifth, the UKB is not nationally representative and is predominantly White, limiting the generalizability of our findings to other racial and ethnic groups. Thus, subgroup analysis by race is primarily able to assess whether the association between early-life sugar restriction and respiratory health is consistent within White individuals, and caution is warranted when extrapolating these findings to non-White groups, which consist of more diverse populations. Sixth, detailed individual-level dietary data were unavailable. Early-life variables, including birth weight, maternal smoking, and breastfeeding, were collected via adult self-report and may be subject to recall error. In particular, breastfeeding status often reflects what participants remember being told by caregivers because it occurs very early in life and usually does not last for a long period. Seventh, using London data as a proxy for nationwide air pollution exposure may not be entirely accurate, but pollution trends across major United Kingdom cities were broadly synchronous during this period [27]. Because London is a representative major city and generally experiences higher pollution levels, this adjustment is likely conservative; nevertheless, our main results remained largely robust. We recognize that our use of median-based PRS grouping may not perfectly reflect the true distribution of genetic risk, and the lack of established clinical thresholds may pose challenges for interpretation.

In summary, our study demonstrates that early-life sugar rationing during the first 1000 d after conception is associated with a reduced risk of asthma and COPD in adulthood, with longer exposure being linked to more pronounced associations and a later onset of disease. Additionally, spirometry results indicated better lung function among those exposed to sugar rationing in early life. These findings underscore the potential long-term relevance of sugar control in utero and infancy for respiratory health and subsequent risk of chronic respiratory diseases. Randomized controlled trials are warranted to verify these findings and develop intervention strategies for the primary prevention of respiratory disorders.

## Author contributions

The authors’ responsibilities were as follows – JZ, CY, FC: conceptualized the study; FC: guarantor; JZ, ZZ, JH: gathered the data; JZ, ZZ, QT, HW, WH, JS: contributed to analyzing and interpreting the data; JZ, ZZ, QY: drafted the initial manuscript; and all authors: participated in the interpretation of the findings and the critical revision of the manuscript, ensuring it contained important intellectual contributions, and read and approved the final version.

## Data availability

The data are accessible through a public, open-access repository. Data from the UKB, ELSA, and HRS are available to researchers on request.

## Code availability

The core statistical analysis code used in this study is publicly available at: https://github.com/benny817817/early-life-sugar-rationing-respiratory-health.

## Funding

This work was supported by the Guangzhou Municipal Research Fund (Z2023209). The study's funding body played no role in the study design, data collection, analysis, interpretation, or manuscript writing, and imposed no restrictions on the publication of the results.

## Conflict of interest

CF reports financial support was provided by RWTH Aachen University. The other authors declare that they have no known competing financial interests or personal relationships that could have appeared to influence the work reported in this article.
