# Supplementary material for: Sugar restriction in the first 1000 days after conception, and long-term respiratory health: a quasi-experiment study
Source: Am J Clin Nutr. 2025 Oct 7;122(6):1759–68. doi: 10.1016/j.ajcnut.2025.09.045 (PMC12799378; doi:10.1016/j.ajcnut.2025.09.045)
Supplement: Multimedia component 1 [file mmc1.docx]

**Supplementary material**

Sugar Restriction in the First 1000 Days after Conception and Long-Term Respiratory Health: A Quasi-experiment Study

Jiazhen Zheng et al.

[**Background** 4](#_Toc204291001)

[**Discussion** 5](#_Toc204291002)

[**eMethods** 6](#_Toc204291003)

[**ELSA** 6](#_Toc204291004)

[**HRS** 6](#_Toc204291005)

[**Details on statistical analysis** 7](#_Toc204291006)

[**Genotyping and quality control** 8](#_Toc204291007)

[**East and North coordinate variables** 10](#_Toc204291008)

[**Mediation analysis** 10](#_Toc204291009)

[**Multiple imputation** 11](#_Toc204291010)

[**Supplementary Table 1.** The numbers (percentages) of participants with missing covariates. 12](#_Toc204291011)

[**Supplementary Table 2.** Assessing the best fitted distribution using the Akaike and Bayesian Information Criterion. 13](#_Toc204291012)

[**Supplementary Table 3.** The effect of sugar rationing on age of disease onset by duration of exposure to rationing. 14](#_Toc204291013)

[**Supplementary Table 4.** Association of early-life sugar rationing exposure with chronic respiratory disease risk after adjustment for black Smoke/PM₁₀ equivalent (London, 1951–1956). 15](#_Toc204291014)

[**Supplementary Table 5.** Association of sugar rationing exposure with risk of chronic respiratory diseases, including only birth and early-life covariates. 16](#_Toc204291015)

[**Supplementary Table 6.** Association of sugar rationing exposure with risk of chronic respiratory diseases, including individuals with prevalent chronic respiratory diseases at baseline (N=6,689). 17](#_Toc204291016)

[**Supplementary Table 7.** Structural equation model fit indices for hypertension, type 2 diabetes and birth weight in mediation analysis of sugar rationing and incident lung disease. 18](#_Toc204291017)

[**Supplementary Table 8.** Baseline data for the contemporaneous control group (UK Biobank), comprising individuals born outside the UK and without exposure to sugar rationing. 19](#_Toc204291018)

[**Supplementary Table 9.** Baseline data for the contemporaneous control group (HRS), comprising individuals without exposure to sugar rationing. 21](#_Toc204291019)

[**Supplementary Table 10.** Baseline characteristics of participants in external validation cohort (ELSA). 22](#_Toc204291020)

[**Supplementary Table 11.** Lung disease risk in participants born between October 1951 and June 1954 versus participants born between July 1954 and March 1956 (UK Biobank contemporaneous control cohort). 24](#_Toc204291021)

[**Supplementary Table 12.** Lung disease risk in participants born between October 1951 and June 1954 versus participants born between July 1954 and March 1956 (HRS Cohort). 25](#_Toc204291022)

[**Supplementary Table 13.** Lung Disease Risk in Rationed and Non-Rationed Participants of the ELSA Cohort. 26](#_Toc204291023)

[**Supplementary Table 14.** Spirometry results for the contemporaneous control group (UK Biobank): participants born between October 1951 and June 1954 versus participants born between July 1954 and March 1956. 27](#_Toc204291024)

[**Supplementary Table 15.** Peak expiratory flow in the contemporaneous control group (HRS): participants born between October 1951 and June 1954 versus participants born between July 1954 and March 1956. 28](#_Toc204291025)

[**Supplementary Table 16.** Spirometry in Rationed and Non-Rationed Participants of the ELSA Cohort. 29](#_Toc204291026)

[**Supplementary Figure 1.** Directed Acyclic Graph. 30](#_Toc204291027)

[**Supplementary Figure 2.** Annual variation of food and food-related variables during the study period. 31](#_Toc204291028)

[**Supplementary Figure 3.** Comparative density distributions of PRS (IPF, COPD and asthma) between rationed and not rationed groups. 32](#_Toc204291029)

[**Supplementary Figure 4.** 33](#_Toc204291030)

[**Supplementary Figure 5.** Comparative density distributions of lung function metrics (FEV1% predicted, FVC% predicted, FEV1/FVC ratio and PEF) between rationed and not rationed groups. 34](#_Toc204291031)

[**Supplementary Figure 6.** Cumulative incidence of chronic respiratory disease between individuals exposed to sugar rationing and never-rationed adults. 35](#_Toc204291032)

[**Supplementary Figure 7.** Hazard ratios for placebo outcomes (influenza [n=208] and herpes zoster [n=154]) by various levels of rationing exposure. 36](#_Toc204291033)

[**Supplementary Figure 8.** The standard mediation analysis investigating the proportion mediated by diabetes, hypertension, and birth weight in the relationship between sugar rationing and lung disease. 37](#_Toc204291034)

[**Supplementary Figure 9.** Hazard ratios for different chronic respiratory diseases by date of birth in individuals born outside of UK and have not experienced sugar rationing (n=5,804). 38](#_Toc204291035)

# **Background**

As highlighted in the United Nations' 2030 Agenda for Sustainable Development, high sugar consumption has become deeply ingrained in global food cultures. ^1^ Amidst growing policy discussions around sugar taxes, ^2^ limiting sugar in infant foods, and regulating related marketing, it has become increasingly important to establish clear connections between early-life sugar exposure and the long-term risk of chronic diseases.^3^ The first 1000 days after conception, from the fetal period to the first two years of life, are a critical window during which nutrition influences lifelong health trajectories. During this period, children often receive excessive sugar intake through maternal diet during pregnancy and breastfeeding, as well as through formula milk and solid foods. ^4^ On average, pregnant and breastfeeding women consume more than 80 grams of added sugar per day, ^5^ which is three times amount recommended by the American Heart Association for women (less than 25 grams per day),^6-7^and most infants frequently consume sugary foods and beverages.^4^

*Study context*

This study utilized a quasi-experimental design based on the UK’s sugar rationing policy. The UK’s sugar rationing policy was in effect from July 1942 until September 1953 and represented a crucial part of the broader wartime food rationing program, which aimed to manage food supplies during World War II and the subsequent period of austerity.^8^ This system relied on scientifically calculated weekly allowances to maintain the minimum nutritional intake required for health, with sugar and sweets strictly limited. During the sugar rationing period, each individual (including pregnant women and children aged five and above) was allocated approximately 8 ounces of sugar per week and 12 ounces of sweets per month, registered with designated retailers. ^7-9^ Children under the age of two were not allocated sugar or sweets as part of their rations. ^10^ The rationing system restricted sugar intake to levels consistent with current dietary guidelines. Adults ingested no more than 40 grams of sugar daily, while children under the age of five consumed fewer than 15 grams per day.

# **Discussion**

Although we observed a numerically stronger association between early-life sugar restriction and respiratory outcomes among individuals whose mothers did not smoke around the time of birth, this interaction did not remain statistically significant after correction for multiple comparisons. However, this trend may implicate potential synergy between dietary and environmental risk factors. Maternal smoking during pregnancy has been shown to increase the risk of asthma, ^11^ wheezing, ^12^ and other respiratory conditions ^13^ in offspring. It contributes to airway inflammation and impaired lung development, ^14^ which may counteract the potential benefits of dietary interventions. Hence, our results may suggest that combined efforts to reduce both sugar intake and harmful exposures such as tobacco could potentially provide greater protection against respiratory diseases in offspring, though further research is warranted to confirm this potential synergy.

Gracner et al. observed that early-life sugar rationing reduced diabetes and hypertension risk by approximately 35% and 20%. ^15^ Our mediation analysis suggested that although **type 2 diabetes** and **hypertension** jointly explain 18% of the sugar rationing–lung disease association, the majority of the association may go beyond the pathways of diabetes and hypertension. Although the exact biological mechanisms are not yet fully understood, several potential mechanisms have been proposed by previous basic researches. Early-life sugar rationing may protect against respiratory diseases by reducing systemic inflammation and oxidative stress, ^16^ which are known to impair lung development. Another proposed mechanism is that limiting sugar intake during early life may help regulate metabolic processes, ^17-19^ such as insulin signaling, which can influence immune system maturation and reduce the risk of allergic and inflammatory responses contributing to asthma and other respiratory conditions.

**eMethods**

## **ELSA**

ELSA is a panel study of a representative cohort of men and women aged ≥50 years living in England. It was designed as a sister study to the Health and Retirement Study in the USA and is multidisciplinary in nature, involving the collection of economic, social, psychological, cognitive, health, biological, and genetic data. The study began in 2002, and the sample has been followed up every two years. The original sample consisted of 11,391 members. Data are collected through computer-assisted personal interviews and self-completion questionnaires, with additional nurse visits for the assessment of biomarkers starting at wave 2 (2004-2005) and every four years thereafter. The baseline of this study was at wave 1 (2002-2003).

Prebronchodilator spirometry testing in the ELSA was performed according to the recommendations of the American Thoracic Society (ATS).^20^ The quality of spirometry testing was evaluated by trained nurses and three successful measurements should be completed for each participant. Spirometry data were collected at wave 2, when lung function measurements were taken. ELSA is harmonized with ageing studies in other countries to facilitate international comparisons and is linked to financial and health registry data. The ELSA cohort was approved by the ethics committee of the London Multi-Centre Research. Informed consent was obtained from each participant.

## **HRS**

The HRS is a longitudinal, nationally representative survey of older US residents, conducted biennially in person with response rates between 70% and 90%.^21^ As the HRS assesses self-reported income and health measures in every survey wave, income data are available for participants both prior to and after 65 years of age, allowing for identification of individuals who were eligible for ACA coverage in midlife and assessment of health outcomes after Medicare eligibility. Additionally, as 94% of respondents currently consent to the linkage of HRS survey data to Medicare enrollment data for all participants and claims data for fee-for-service beneficiaries, it is possible to assess key utilization outcomes after Medicare entry.^22^ The breathing test was conducted in Wave 8. The study was approved by the University of Michigan institutional review board without the need for informed consent due to use of deidentified data and followed the Strengthening the Reporting of Observational Studies in Epidemiology ([STROBE](http://www.equator-network.org/reporting-guidelines/strobe/)) guidelines for cross-sectional and cohort studies.

## **Details on statistical analysis**

We employed an event study design, estimated using multivariate parametric hazard models, to calculate hazard ratios (HRs) for respiratory outcomes in individuals exposed to rationing compared to those unexposed. The following equation was estimated using parametric hazard models: $h\left( t \right)_{i}=\mathrm{ex}p \left( \sum_{k=-3,k\neq0}^{5} \beta_{k}\cdot\left( \text{Birthgroup}_{k}=1 \right)+\theta^{'}X_{i}+\mu_{m}+\delta_{t} \right)\cdot exp \left( \gamma t \right).$ In this model, h(t) represents the hazard rate at time t, and γ is the shape parameter of the Gompertz distribution. The subscript k indicates the event periods relative to the reference period (July 1954 to March 1956, k=0), which corresponds to the birth months of adults who were the first group not exposed to sugar rationing. The values k=1 to k=5 represent 6-month intervals of rationing exposure (9 months if in utero), categorized by birth months as follows: in utero (born between October 1951 and March 1952, k=1), in utero and up to age 6 months (born between April 1952 and September 1952, k=2), in utero and up to age 12 months (born between October 1952 and March 1953, k=3), in utero and up to age 18 months (born between April 1953 and September 1953, k=4), and in utero and up to age 24 months (born between October 1953 and June 1954, k=5). The subscripts k=-1 to k=-3 represent adults born after rationing ended, specifically between January 1955 and June 1955 (k=-1), July 1955 to December 1955 (k=-2), and January 1956 to March 1956 (k=-3).

This model compared hazard rates across different periods with the hazard rate of the reference group—adults born between July and December 1954, who were not exposed to rationing. The relative differences in hazard rates were represented by the coefficients (β) for each period (k), expressed as hazard ratios (HRs). These coefficients, along with their 95% confidence intervals, were presented in Figure 2, adjusted for serial correlation in the outcome by accounting for the birth year-month.

All models incorporated time-invariant individual-level variables (denoted by 𝜃𝑋), including age, sex. race, birth location, calendar month of birth, household income, Townsend deprivation index, real food prices (adjusted for the consumer price index), parental lung disease, whether parents still alive, maternal smoking around birth, whether breastfed as a baby, the social average fat intake around the period of birth (derived from the NSF), baseline lifestyle factors (smoking status, alcohol intake and physical activity), baseline medical conditions (CVD, hypertension, diabetes, digestive disease, kidney disease, and liver disease) and survey year. Calendar month of birth (with January as the reference category) was included to adjust for seasonal effects (denoted by 𝜇), while fixed effects for the survey calendar year (denoted by 𝛿) were used to account for nationwide secular trends in the outcomes.

Binary indicators for the birth timing of non-rationed adults were included for two main reasons: first, to provide a comparison group consisting of adults born after the end of rationing; and second, to examine outcome differences across adults born in 6-month intervals. This approach allowed us to address the potential bias from general time trends or advancements in disease diagnostics that could result in earlier detection of diseases among non-rationed adults. If such biases were present, chronic disease risk would likely increase over time. Our key assumption was that, after accounting for control variables, the disease risk for non-rationed adults born after December 1954 would be similar, with their HRs remaining around 1.

In addition, we grouped birth cohorts according to three biologically and nutritionally relevant windows: (1) in utero only—capturing the impact of gestational exposure during critical periods of lung and immune development;^23^ (2) in utero plus up to age 1, including both prenatal and infant stages when postnatal lung growth and immune maturation occur;^24^ and (3) in utero plus 1–2 years, representing exposure throughout the first 1,000 days, a widely recognized window for developmental programming of long-term disease risk.^25^ These divisions align with the DOHaD paradigm and are supported by epidemiological and experimental evidence linking nutritional restriction during these periods to persistent effects on respiratory health. The formular is: $h\left( t \right)_{i}=\mathrm{ex}p \left( \sum_{k=1}^{3} \beta_{k}\cdot\left( \text{Birthgroup}_{k} \right)+\theta^{'}X_{i}+\mu_{m}+\delta_{t} \right)\cdot exp \left( \gamma t \right).$ We developed categorical variables with values ranging from 0 to 3. We compared non-rationed adults (the reference group, consisting of individuals born after July 1954, k = 0) with those exposed to rationing during different life stages: in utero (k = 1), in utero and up to age 1 (k = 2), or in utero plus 1-2 years (k = 3) **(see Table 2)**.

## **Genotyping and quality control**

UK Biobank samples (version 3; March 2018) were genotyped for > 800,000 SNPs using either the Affymetrix UK BiLEVE Axiom array or the Affymetrix UK Biobank Axiom array. Imputation via IMPUTE2 was carried out centrally by UK Biobank researchers using the merged 1000 Genomes Project panel and UK 10K panel.^26^ After imputation, variant-level quality control (QC) was performed by filtering SNPs on two criteria: (1) minor allele frequency < 0.01 and (2) imputation quality score < 0.3. A total of 9,505,768 imputed autosomal SNPs passed the QC criteria. PRS were constructed to quantify the cumulative genetic predisposition for each participant. SNP effect sizes were derived from genome-wide association study (GWAS) summary statistics, in which association analyses were performed using BOLT-LMM,^27^ a linear mixed model that jointly models all SNPs and accounts for population structure as well as linkage disequilibrium (LD). All GWAS analyses were adjusted for relevant covariates, including the top principal components (to control for ancestry), age, sex, and genotyping array.

To generate the PRS, we applied LD clumping using PLINK version 1.9 to select independent SNPs.^28^ The clumping procedure used an r² < 0.1 threshold within a 1,000-kb window, thereby minimizing the inclusion of correlated variants and reducing redundancy in the risk score. For each individual, the PRS was calculated as the sum of risk alleles weighted by their GWAS-derived joint effect sizes. Where available, we used joint effect estimates from BOLT-LMM to ensure that each SNP’s contribution to the PRS reflected its independent association with the trait, accounting for LD structure.

A total of 23 SNPs for IPF,^29^ 81 SNPs for COPD^30^ and 125 SNPs for asthma^31^ were kept for PRS construction. The count of alleles (0, 1, or 2) per individual was summed after being multiplied by the effect size pertaining to the association between the specific SNP and the respective disease. PRS was calculated using the formula: PRS=β_1_×SNP_1_+β_2_×SNP_2_+…+β_n_ ×SNP_n_. SNP_i_ represents the risk allele number of each SNP.^32^ A higher PRS suggests a greater genetic susceptibility to the disease. Participants with PRS scores in the lowest quintile were classified as "low", those in the second to fourth quintiles as "medium", and those in the highest quintile as "high". Given that certain studies identified multiple related variants within the same genomic region, independent SNPs were chosen based on the lowest reported p value. All procedures were conducted according to established protocols and recommended practices in the field.^33-34^

## **East and North coordinate variables**

UK Biobank recorded participants’ birth locations using "East" and "North" coordinates based on the British National Grid reference system (OSGB1936, EPSG:27700), which provides precise geolocation in meters rather than latitude and longitude. These coordinates were derived from participants’ reported birth towns or districts in England, Scotland, or Wales, and represent their easting and northing relative to a national reference point. This system enables spatial analysis of participants’ birthplaces across the UK, supporting geographic stratification and adjustment for potential regional variation in epidemiological studies.

## **Mediation analysis**

We investigated whether type 2 diabetes, hypertension, and birth weight mediated the association between early-life sugar rationing (exposure, X) and incident lung disease (outcome, Y; defined as the occurrence of any of IPF, COPD, or asthma).

Mediators were identified as type 2 diabetes and hypertension diagnosed before the onset of lung disease, ascertained using self-reported questionnaires (UK Biobank data fields 2443 and 1065) and diagnostic records from primary care and hospital databases (data fields 130708, 131286, and 131294). Diagnoses were based on ICD-10 codes (E11 for type 2 diabetes; I10/I15 for hypertension). To perform these analyses, we collected data on 3,917 cases of diabetes and 18,678 cases of hypertension that occurred before the onset of 10,250 lung disease cases, of which 3,248 arose during follow-up.

**Primary mediation analyses were performed using structural equation modeling (SEM)** via the lavaan package in R.^35^ SEM enables simultaneous estimation of the direct and indirect (mediated) effects within a single model and allows inclusion of multiple mediators (diabetes, hypertension, and birth weight) in parallel. This approach overcomes several limitations of traditional stepwise methods and provides robust,^36^ model-based estimates with confidence intervals. SEM models included the same set of covariates as Model 2, as specified in the main analysis. Model fit was evaluated using established indices: RMSEA (Root Mean Square Error of Approximation), CFI (Comparative Fit Index), TLI (Tucker-Lewis Index), and SRMR (Standardized Root Mean Square Residual), with thresholds of RMSEA < 0.05, CFI/TLI > 0.95, and SRMR < 0.05 considered indicative of good fit.^37^

All mediation models were restricted to cases where mediator onset preceded the diagnosis of lung disease to ensure temporal ordering. The proportion of the total effect of sugar rationing on lung disease risk mediated by each factor was estimated.

## **Multiple imputation**

We utilized the ‘mcar_test’ function from the ‘naniar’ R package to assess the Missing at Random assumption.^38^ No statistically significant evidence against the null hypothesis (p > 0.05) was found, suggesting that the missingness could reasonably be considered completely at random. Additionally, the missing data patterns were less than 5%, indicating that missingness is assumed not to influence the analysis.

Upon examination, we found that continuous variables adhere to a normal distribution, and no significantly unbalanced categorical variables were identified. Given that the variable types of the missing values in our study are mixed, and considering that ‘mice_pmm’ (Predictive mean matching) is a less restrictive method compared to Bayesian linear regression, Logistic regression, Polytomous logistic regression, and Proportional odds model, we opted to use the ‘mice_pmm’ package for imputing missing values. We set the caliper at 5%.

This approach aligns with common practices in UK Biobank-based studies. The method we employed is consistent with the default recommendation from the ‘mice’ package. In total, we imputed twenty datasets, incorporating all variables utilized in the analysis. Notably, we observed no significant deviations between the imputed and observed values.

**Supplementary Table 1.** The numbers (percentages) of participants with missing covariates.

| Covariates | n | % |
| --- | --- | --- |
| Household income | 5374 | 9.16 |
| Parental health information | 3003 | 5.12 |
| Physical activity | 316 | 0.54 |
| BMI | 234 | 0.40 |
| Ethnicity | 193 | 0.33 |
| Smoking status | 176 | 0.30 |
| Breastfed as a baby | 88 | 0.15 |
| Townsend Deprivation Index | 53 | 0.09 |

**Supplementary Table 2.** Assessing the best fitted distribution using the Akaike and Bayesian Information Criterion.

|  | Gompertz | Weibull | Logistic | Log normal | Exponential |
| --- | --- | --- | --- | --- | --- |
| IPF |  |  |  |  |  |
| AIC | 10244.13 | 10276.84 | 10300.34 | 10300.91 | 10394.17 |
| BIC | 10258.77 | 10294.80 | 10318.3 | 10318.87 | 10403.15 |
| COPD |  |  |  |  |  |
| AIC | 47683.04 | 47701.34 | 48188.52 | 47779.93 | 48036.27 |
| BIC | 47696.20 | 47719.30 | 48206.48 | 47797.89 | 48045.25 |
| Asthma |  |  |  |  |  |
| AIC | 44709.91 | 44729.75 | 45758.18 | 44752.48 | 44729.99 |
| BIC | 44721.58 | 44747.71 | 45776.14 | 44770.44 | 44738.97 |

Based on this criterion we proceeded with Gompertz distribution as our preferred one. AIC = Akaike information criterion; BIC = Bayesian information criterion. IPF = idiopathic pulmonary fibrosis; COPD = chronic obstructive pulmonary disease.

**Supplementary Table 3.** The effect of sugar rationing on age of disease onset by duration of exposure to rationing.

|  | Not Rationed | In utero | In utero + (0, 1] year | In utero + (1, 2] year |
| --- | --- | --- | --- | --- |
| IPF |  |  |  |  |
| Age of onset (95%CI) | 62.78 (62.04, 63.52) | 64.62 (63.75, 65.49) | 64.87 (64.13, 65.60) | 65.52 (64.74, 66.30) |
| Delay in age of onset (years) (95% CI) | Reference | 1.83 (0.71, 2.96) | 2.09 (1.05, 3.12) | 2.74 (1.67, 3.80) |
| COPD |  |  |  |  |
| Age of onset (95%CI) | 62.13 (61.84, 62.42) | 62.93 (62.54, 63.32) | 63.79 (63.45, 64.13) | 64.98 (64.61, 65.34) |
| Delay in age of onset (years) (95% CI) | Reference | 0.80 (0.32, 1.28) | 1.66 (1.22, 2.11) | 2.85 (2.39, 3.31) |
| Asthma |  |  |  |  |
| Age of onset (95%CI) | 60.12 (59.81, 60.43) | 61.54 (61.05, 62.03) | 62.48 (62.07, 62.89) | 63.74 (63.33, 64.15) |
| Delay in age of onset (years) (95% CI) | Reference | 1.42 (0.84, 1.99) | 2.36 (1.85, 2.87) | 3.62 (3.11, 4.13) |

Time-to-event models assume Gompertz distribution were used. IPF = idiopathic pulmonary fibrosis; COPD = chronic obstructive pulmonary disease.

**Supplementary Table 4.** Association of early-life sugar rationing exposure with chronic respiratory disease risk after adjustment for black Smoke/PM₁₀ equivalent (London, 1951–1956).

|  | Not Rationed | In utero | In utero + (0, 1] year | In utero + (1, 2] year | P for trend |
| --- | --- | --- | --- | --- | --- |
| IPF |  |  |  |  |  |
| Total cases/Total sample size | 120/21516 | 62/9660 | 87/13630 | 86/13864 |  |
| Person-years | 299015 | 133919 | 188680 | 191308 |  |
| HR (95%CI); Model 3 plus Black smoke/PM₁₀ equivalent | Reference | 1.00 (0.72-1.40) | 0.87 (0.60-1.24) | 0.75 (0.48-1.16) | 0.167 |
| COPD |  |  |  |  |  |
| Total cases/Total sample size | 685/21516 | 317/9660 | 438/13630 | 411/13864 |  |
| Person-years | 296336 | 132582 | 186804 | 188894 |  |
| HR (95%CI); Model 3 plus Black smoke/PM₁₀ equivalent | Reference | 0.94 (0.81-1.09) | 0.86 (0.74-1.00) | 0.76 (0.65-0.91) | 0.008 |
| Asthma |  |  |  |  |  |
| Total cases/Total sample size | 632/21516 | 273/9660 | 409/13630 | 400/13864 |  |
| Person-years | 294432 | 131975 | 186084 | 188689 |  |
| HR (95%CI); Model 3 plus Black smoke/PM₁₀ equivalent | Reference | 0.88 (0.76-1.02) | 0.85 (0.73-1.01) | 0.77 (0.63-0.93) | 0.010 |

Model 3 adjusted for age, sex, race, birth location, calendar month of birth, household income, Townsend deprivation index, real food prices (adjusted for the consumer price index), parental lung disease, whether parents still alive, maternal smoking around birth, whether breastfed as a baby and the social average fat intake around the period of birth (derived from the NSF), baseline lifestyle factors (smoking status, alcohol intake and physical activity), baseline medical conditions (CVD, hypertension, diabetes, digestive disease, kidney disease, and liver disease) and survey year. HRs and 95% confidence intervals were estimated using Cox proportional hazards models (Model 3) additionally adjusted for annual mean black smoke/PM₁₀ equivalent concentrations. Black smoke/PM₁₀ equivalent values were derived from annual mean concentrations measured in London, based on data published by Jacqueline CK Lam et al.,^39^ and were used as a proxy for ambient air pollution exposure.

**Supplementary Table 5.** Association of sugar rationing exposure with risk of chronic respiratory diseases, including only birth and early-life covariates.

|  | Not Rationed | In utero | In utero + (0, 1] year | In utero + (1, 2] year | P for trend |
| --- | --- | --- | --- | --- | --- |
| IPF |  |  |  |  |  |
| Total cases/Total sample size | 120/21516 | 62/9660 | 87/13630 | 86/13864 |  |
| Person-years | 299015 | 133919 | 188680 | 191308 |  |
| HR (95%CI) | Reference | 0.97 (0.69-1.36) | 0.85 (0.58-1.21) | 0.73 (0.47-1.14) | 0.160 |
| COPD |  |  |  |  |  |
| Total cases/Total sample size | 685/21516 | 317/9660 | 438/13630 | 411/13864 |  |
| Person-years | 296336 | 132582 | 186804 | 188894 |  |
| HR (95%CI) | Reference | 0.90 (0.78-1.04) | 0.82 (0.70-0.96) | 0.72 (0.61-0.87) | <0.001 |
| Asthma |  |  |  |  |  |
| Total cases/Total sample size | 632/21516 | 273/9660 | 409/13630 | 400/13864 |  |
| Person-years | 294432 | 131975 | 186084 | 188689 |  |
| HR (95%CI) | Reference | 0.87 (0.75-1.01) | 0.84 (0.71-0.99) | 0.73 (0.59-0.90) | 0.003 |

Covariates included: age, sex, race, birth location, calendar month of birth, real food prices (adjusted for the consumer price index), parental lung disease, whether parents were still alive, maternal smoking around birth, whether breastfed as a baby, the social average fat intake around the period of birth (derived from NSF data), and survey year. HR = hazard ratio; 95% CI = 95% confidence interval.
Only variables related to birth or early-life exposure were included in the Cox regression models.

**Supplementary Table 6.** Association of sugar rationing exposure with risk of chronic respiratory diseases, including individuals with prevalent chronic respiratory diseases at baseline (N=6,689).

|  | Not Rationed | In utero | In utero + (0, 1] year | In utero + (1, 2] year | P for trend |
| --- | --- | --- | --- | --- | --- |
| IPF |  |  |  |  |  |
| Total cases/Total sample size | 238/21516 | 115/9660 | 163/13630 | 161/13864 |  |
| OR (95%CI) | Reference | 0.98 (0.68-1.28) | 0.85 (0.60-1.10) | 0.78 (0.54-1.16) | 0.109 |
| COPD |  |  |  |  |  |
| Total cases/Total sample size | 1467/21516 | 670/9660 | 927/13630 | 875/13864 |  |
| OR (95%CI) | Reference | 0.92 (0.81-1.03) | 0.85 (0.76-0.96) | 0.76 (0.66-0.87) | 0.003 |
| Asthma |  |  |  |  |  |
| Total cases/Total sample size | 1340/21516 | 575/9660 | 851/13630 | 842/13864 |  |
| OR (95%CI) | Reference | 0.89 (0.78-1.00) | 0.85 (0.73-0.97) | 0.75 (0.63-0.88) | 0.006 |

Covariates included: age, sex, race, birth location, calendar month of birth, real food prices (adjusted for the consumer price index), parental lung disease, whether parents were still alive, maternal smoking around birth, whether breastfed as a baby, the social average fat intake around the period of birth (derived from NSF data), and survey year. OR = odds ratio; 95% CI = 95% confidence interval.
Logistic regression models were used. All individuals, including those with prevalent chronic respiratory diseases at baseline, were included in the analysis.

# **Supplementary Table 7.** Structural equation model fit indices for hypertension, type 2 diabetes and birth weight in mediation analysis of sugar rationing and incident lung disease.

| SEM Model | RMSEA | CFI | TLI | SRMR |
| --- | --- | --- | --- | --- |
| Model with incident hypertension | 0.028 | 0.98 | 0.97 | 0.022 |
| Model with incident type 2 diabetes | 0.026 | 0.97 | 0.96 | 0.021 |
| Model with birth weight | 0.038 | 0.97 | 0.96 | 0.030 |

Model fit indices (RMSEA < 0.05, CFI > 0.95, TLI > 0.95, SRMR < 0.05) indicated excellent fit for structural equation models (SEM) with incident hypertension, type 2 diabetes and birth weight.

RMSEA = Root Mean Square Error of Approximation; CFI = Comparative Fit Index; TLI = Tucker-Lewis Index; SRMR = Standardized Root Mean Square Residual; SEM = structural equation modeling.

**Supplementary Table 8.** Baseline data for the contemporaneous control group (UK Biobank), comprising individuals born outside the UK and without exposure to sugar rationing.

|  | Total | Born between July 1954 and March 1956 | Born between October 1951 and June 1954 | P value |
| --- | --- | --- | --- | --- |
| No. of participants | 5804 | 2262 | 3542 |  |
| Age at entry, mean (SD), years | 54.8 (1.6) | 53.4 (1.1) | 55.7 (1.2) | <0.001 |
| Women | 3421 (58.9) | 1319 (58.3) | 2102 (59.3) | 0.451 |
| Birth month |  |  |  |  |
| Mar 1-May 31 | 1377 (23.7) | 420 (18.6) | 957 (27.0) | <0.001 |
| Jun 1-Aug 31 | 1268 (21.8) | 515 (22.8) | 753 (21.3) |  |
| Sep 1-Nov 30 | 1498 (25.8) | 665 (29.4) | 833 (23.5) |  |
| Dec 1-Feb 28 | 1661 (28.6) | 662 (29.3) | 999 (28.2) |  |
| White, % | 2500 (43.1) | 902 (39.9) | 1598 (45.1) | <0.001 |
| Education, % |  |  |  |  |
| Below A levels | 2096 (36.1) | 813 (35.9) | 1283 (36.2) | 0.373 |
| A levels | 1253 (21.6) | 470 (20.8) | 783 (22.1) |  |
| College/university | 1564 (26.9) | 636 (28.1) | 928 (26.2) |  |
| Professional/other | 891 (15.4) | 343 (15.2) | 548 (15.5) |  |
| Household income, £ |  |  |  |  |
| <18,000 | 2088 (36.0) | 806 (35.6) | 1282 (36.2) | 0.548 |
| 18,000-30,999 | 1014 (17.5) | 392 (17.3) | 622 (17.6) |  |
| 31,000-51,999 | 1146 (19.7) | 464 (20.5) | 682 (19.3) |  |
| 52,000-100,000 | 1038 (17.9) | 412 (18.2) | 626 (17.7) |  |
| >100,000 | 518 (8.9) | 188 (8.3) | 330 (9.3) |  |
| Townsend deprivation index, mean (SD) | 0.3 (3.5) | 0.4 (3.5) | 0.3 (3.5) | 0.118 |
| Smoking status, % |  |  |  |  |
| Current | 669 (11.5) | 274 (12.1) | 395 (11.2) | 0.045 |
| Never | 3515 (60.6) | 1397 (61.8) | 2118 (59.8) |  |
| Previous | 1620 (27.9) | 591 (26.1) | 1029 (29.1) |  |
| Pack-years of smoking, mean (SD) | 18.9 (14.6) | 17.6 (13.2) | 19.6 (15.4) | 0.015 |
| Alcohol consumption frequency, times/wk |  |  |  |  |
| <3 | 4170 (71.8) | 1632 (72.1) | 2538 (71.7) | 0.705 |
| >=3 | 1634 (28.2) | 630 (27.9) | 1004 (28.3) |  |
| BMI, mean (SD), kg/m2 | 27.5 (5.0) | 27.5 (5.3) | 27.5 (4.9) | 0.814 |
| Summed MET minutes per week for all activity, IQR | 2490.9 (2318.2) | 2551.6 (2320.1) | 2452.1 (2316.5) | 0.111 |
| Personal medical condition |  |  |  |  |
| Cardiovascular disease | 282 (4.9) | 100 (4.4) | 182 (5.1) | 0.239 |
| Hypertension | 3052 (52.6) | 1144 (50.6) | 1908 (53.9) | 0.015 |
| High cholesterol | 1057 (18.2) | 390 (17.2) | 667 (18.8) | 0.135 |
| Diabetes | 349 (6.0) | 140 (6.2) | 209 (5.9) | 0.693 |
| Digestive disease | 71 (1.2) | 20 (0.9) | 51 (1.4) | 0.079 |
| Kidney disease | 164 (2.8) | 60 (2.7) | 104 (2.9) | 0.579 |
| Liver disease | 249 (4.3) | 97 (4.3) | 152 (4.3) | 0.993 |
| Parents' condition |  |  |  |  |
| Parents diagnosed with lung disease | 736 (12.7) | 273 (12.1) | 463 (13.1) | 0.281 |
| Parents still alive | 906 (15.6) | 432 (19.1) | 474 (13.4) | <0.001 |
| Maternal smoking around birth | 694 (12.0) | 257 (11.4) | 437 (12.3) | 0.282 |
| Breastfed as a baby | 4179 (72.0) | 1672 (73.9) | 2507 (70.8) | 0.010 |
| Birth weight, mean (SD), kg | 3.3 (0.4) | 3.2 (0.5) | 3.3 (0.4) | 0.268 |
| FEV1 % predicted, mean (SD) | 84.7 (23.3) | 84.9 (23.3) | 84.6 (23.3) | 0.622 |
| FVC % predicted, mean (SD) | 88.7 (24.3) | 88.9 (24.6) | 88.6 (24.1) | 0.597 |
| FEV1/FVC ratio | 0.8 (0.1) | 0.8 (0.1) | 0.8 (0.1) | 0.055 |
| PEF, mean (SD), litres/min | 383.2 (132.5) | 384.7 (131.9) | 382.4 (134.7) | 0.689 |

Data are presented as No. (%) or mean (SD). BMI = body mass index; MET = metabolic equivalent of task; FEV1 = forced expiratory volume in 1 second; FVC = forced vital capacity; PEF = peak expiratory flow. * P values were obtained from either a chi-square test or a Mann-Whitney U test, comparing differences between participants born during these two periods.

**Supplementary Table 9.** Baseline data for the contemporaneous control group (HRS), comprising individuals without exposure to sugar rationing.

|  | Overall | Born between July 1954 and March 1956 | Born between October 1951 and June 1954 | P |
| --- | --- | --- | --- | --- |
| Participants | 2995 | 1497 | 1498 |  |
| Age (mean (SD)) | 43.8(1.6) | 42.6(1.3) | 44.9(0.9) | <0.001 |
| Sex (%) |  |  |  |  |
| Female | 1649(55.1) | 829(55.4) | 820(54.7) | 0.753 |
| Male | 1346(44.9) | 668(44.6) | 678(45.3) |  |
| Race (%) |  |  |  |  |
| Non-white | 1209(40.4) | 618(41.3) | 591(39.5) | 0.325 |
| White | 1786(59.6) | 879(58.7) | 907(60.5) |  |
| Education (%) |  |  |  |  |
| Below high school | 479(16.0) | 246(16.4) | 233(15.6) | 0.740 |
| College or above | 1631(54.5) | 806(53.8) | 825(55.1) |  |
| High school | 885(29.5) | 445(29.7) | 440(29.4) |  |
| Marital status (%) |  |  |  |  |
| Married or partnered | 2930(97.8) | 1463(97.7) | 1467(97.9) | 0.809 |
| Never married | 7(0.2) | 3(0.2) | 4(0.3) |  |
| Separated/divorced/Widowed | 58(1.9) | 31(2.1) | 27(1.8) |  |
| Total Wealth (mean (SD)), £ | 215857.9(399855.7) | 209417.8(378205.2) | 222293.7(420407.7) | 0.378 |
| Smoking status (%) |  |  |  |  |
| Ever smokers | 1517(50.7) | 701(46.8) | 816(54.5) | <0.001 |
| Never smokers | 1478(49.3) | 796(53.2) | 682(45.5) |  |
| Drinking status (%) |  |  |  |  |
| Ever drinkers | 1926(64.3) | 995(66.5) | 931(62.1) | 0.015 |
| Never drinkers | 1069(35.7) | 502(33.5) | 567(37.9) |  |
| Physical activity (%) |  |  |  |  |
| Vigorous activity 3+/wk | 1501(50.1) | 796(53.2) | 705(47.1) | 0.001 |
| Vigorous activity less than 3/wk | 1494(49.9) | 701(46.8) | 793(52.9) |  |
| Hypertension (%) |  |  |  |  |
| No | 2598(86.7) | 1302(87.0) | 1296(86.5) | 0.752 |
| Yes | 397(13.3) | 195(13.0) | 202(13.5) |  |
| Diabetes (%) |  |  |  |  |
| No | 2883(96.3) | 1445(96.5) | 1438(96.0) | 0.502 |
| Yes | 112(3.7) | 52(3.5) | 60(4.0) |  |
| Cardiovascular disease (%) |  |  |  |  |
| No | 2949(98.5) | 1472(98.3) | 1477(98.6) | 0.654 |
| Yes | 46(1.5) | 25(1.7) | 21(1.4) |  |
| Peak expiratory flow,  litres per minute (mean (SD)) | 420.6(121.3) | 419.2(113.9) | 422.1(128.2) | 0.520 |

Data are presented as No. (%) or mean (SD). * P values were obtained from either a chi-square test or a Mann-Whitney U test, comparing differences between participants born during these two periods.

**Supplementary Table 10.** Baseline characteristics of participants in external validation cohort (ELSA).

|  | Overall | Non-rationed | Rationed | P |
| --- | --- | --- | --- | --- |
| Participants | 1900 | 842 | 1058 |  |
| Age (mean (SD)) | 48.2 (1.7) | 46.9 (1.5) | 49.3 (1.0) | <0.001 |
| Sex (%) |  |  |  |  |
| Female | 1081 (56.9) | 493 (58.6) | 588 (55.6) | 0.210 |
| Male | 819 (43.1) | 349 (41.4) | 470 (44.4) |  |
| Race (%) |  |  |  |  |
| Non-white | 110 (5.8) | 49 (5.8) | 61 (5.8) | 0.996 |
| White | 1790 (94.2) | 793 (94.2) | 997 (94.2) |  |
| Education (%) |  |  |  |  |
| Below high school | 438 (23.1) | 191 (22.7) | 247 (23.3) | 0.190 |
| College or above | 895 (47.1) | 388 (46.1) | 507 (47.9) |  |
| High school | 442 (23.3) | 214 (25.4) | 228 (21.6) |  |
| Other | 125 (6.6) | 49 (5.8) | 76 (7.2) |  |
| Marital status (%) |  |  |  |  |
| Married or partnered | 1872 (98.5) | 835 (99.2) | 1037 (98.0) | 0.026 |
| Never married | 5 (0.3) | 3 (0.4) | 2 (0.2) |  |
| Separated/divorced/Widowed | 23 (1.2) | 4 (0.5) | 19 (1.8) |  |
| Family wealth (mean (SD)), £ | 220846.8 (276497.2) | 225367.4 (295155.4) | 217249.1 (260782.3) | 0.525 |
| Physical activity (%) |  |  |  |  |
| > 1 per week | 616 (32.4) | 260 (30.9) | 356 (33.6) | <0.001 |
| 1 per week | 113 (5.9) | 31 (3.7) | 82 (7.8) |  |
| 1-3 per mon | 194 (10.2) | 81 (9.6) | 113 (10.7) |  |
| hardly ever or never | 977 (51.4) | 470 (55.8) | 507 (47.9) |  |
| Smoking status (%) |  |  |  |  |
| Ever smokers | 1241 (65.3) | 552 (65.6) | 689 (65.1) | 0.881 |
| Never smokers | 659 (34.7) | 290 (34.4) | 369 (34.9) |  |
| Drinking status (%) |  |  |  |  |
| Ever drinkers | 1819 (95.7) | 802 (95.2) | 1017 (96.1) | 0.410 |
| Never drinkers | 81 (4.3) | 40 (4.8) | 41 (3.9) |  |
| Hypertension (%) |  |  |  |  |
| No | 1482 (78.0) | 674 (80.0) | 808 (76.4) | 0.062 |
| Yes | 418 (22.0) | 168 (20.0) | 250 (23.6) |  |
| Diabetes (%) |  |  |  |  |
| No | 1879 (98.9) | 832 (98.8) | 1047 (99.0) | 0.932 |
| Yes | 21 (1.1) | 10 (1.2) | 11 (1.0) |  |
| CVD (%) |  |  |  |  |
| No | 1823 (95.9) | 800 (95.0) | 1023 (96.7) | 0.084 |
| Yes | 77 (4.1) | 42 (5.0) | 35 (3.3) |  |
| Peak expiratory flow,  litres per minute (mean (SD)) | 469.3 (119.1) | 467.0 (118.7) | 471.2 (119.5) | 0.451 |
| FVC, litre (mean (SD)) | 3.8 (1.0) | 3.7 (1.0) | 3.8 (1.0) | 0.122 |
| FEV1, liter (mean (SD)) | 2.9 (0.7) | 2.9 (0.7) | 2.9 (0.7) | 0.945 |

Data are presented as No. (%) or mean (SD). FEV1 = forced expiratory volume in 1 second; FVC = forced vital capacity. * P values were obtained from either a chi-square test or a Mann-Whitney U test comparing difference between rationed and not rationed group.

**Supplementary Table 11.** Lung disease risk in participants born between October 1951 and June 1954 versus participants born between July 1954 and March 1956 (UK Biobank contemporaneous control cohort).

|  | **IPF** | **COPD** | **Asthma** |
| --- | --- | --- | --- |
| Event (n)/Person-years in participants born between July 1954 and March 1956 | 13/30843 | 43/30761 | 49/29495 |
| Event (n)/Person-years in participants born between October 1951 and June 1954 | 24/48100 | 97/47778 | 75/45931 |
| Model 1 HR (95% CI) | 0.92 (0.59-1.80) | 1.11 (0.68-1.82) | 1.05 (0.75-1.55) |
| P for model 1 | 0.497 | 0.670 | 0.577 |
| Model 2 HR (95% CI) | 0.95 (0.60-1.88) | 1.06 (0.65-1.74) | 1.09 (0.77-1.61) |
| P for model 2 | 0.531 | 0.795 | 0.664 |
| Model 3 HR (95% CI) | 0.97 (0.62-1.92) | 1.04 (0.63-1.71) | 1.06 (0.75-1.58) |
| P for Model 3 | 0.554 | 0.864 | 0.619 |

The HR for lung disease risk was derived by comparing participants born between October 1951 and June 1954 to those born between July 1954 and March 1956. Parametric hazard models based on the Gompertz distribution were used. Model 1 adjusted for age and sex. Model 2 additionally adjusted for race (white vs not), household income, Townsend deprivation index, smoking status, alcohol consumption, personal medical condition (cardiovascular disease, hypertension, diabetes, digestive disease, kidney disease and liver disease), parental lung disease, maternal smoking around birth, and whether breastfed as a baby. Model 3 adjusted for terms in Model 2 and calendar month of birth and survey year.

**Supplementary Table 12.** Lung disease risk in participants born between October 1951 and June 1954 versus participants born between July 1954 and March 1956 (HRS Cohort).

| Event (n)/Person-years in participants born between July 1954 and March 1956 | 198/25850 |
| --- | --- |
| Event (n)/Person-years in participants born between October 1951 and June 1954 | 203/25320 |
| Model 1 HR (95% CI) | 1.14 (0.86-1.52) |
| P for model 1 | 0.341 |
| Model 2 HR (95% CI) | 1.05 (0.79-1.39) |
| P for model 2 | 0.725 |
| Model 3 HR (95% CI) | 1.06 (0.80-1.42) |
| P for Model 3 | 0.649 |

The HR for lung disease risk was derived by comparing participants born between October 1951 and June 1954 to those born between July 1954 and March 1956. Parametric hazard models based on the Gompertz distribution were used. Model 1 adjusts for age, sex, and race. Model 2 adjusts for model 1 plus education, marital status, total wealth, smoking status, drinking status, and physical activity. Model 3 adjusts for model 2 plus hypertension, diabetes, and cardiovascular disease. HR = hazard ratio; CI = confidence interval.

**Supplementary Table 13.** Lung Disease Risk in Rationed and Non-Rationed Participants of the ELSA Cohort.

| Event (n)/Person-years in non-rationed participants | 98/15002 |
| --- | --- |
| Event (n)/Person-years in rationed participants | 77/18698 |
| Rationed vs not rationed--Model 1 HR (95% CI) | 0.91 (0.59-1.40) |
| P for model 1 | 0.687 |
| Rationed vs not rationed--Model 2 HR (95% CI) | 0.87 (0.56-1.34) |
| P for model 2 | 0.535 |
| Rationed vs not rationed--Model 3 HR (95% CI) | 0.86 (0.56-1.32) |
| P for Model 3 | 0.509 |

Parametric hazard models based on the Gompertz distribution were used. Model 1 adjusts for age, sex, and race. Model 2 adjusts for model 1 plus education, marital status, family wealth, smoking status, drinking status, and physical activity. Model 3 adjusts for model 2 plus hypertension, diabetes, and cardiovascular disease. HR = hazard ratio; CI = confidence interval.

**Supplementary Table 14.** Spirometry results for the contemporaneous control group (UK Biobank): participants born between October 1951 and June 1954 versus participants born between July 1954 and March 1956.

| Spirometry | Coefficient (95% CI)  for model 1 | P Value  for model 1 | Coefficient (95% CI)  for model 2 | P Value  for model 2 | Coefficient (95% CI)  for model 3 | P Value  for model 3 |
| --- | --- | --- | --- | --- | --- | --- |
| FEV1 % predicted | 0.635 (-1.096, 2.367) | 0.392 | 0.124 (-1.372, 1.621) | 0.871 | 0.255 (-1.216, 1.725) | 0.734 |
| FVC % predicted | 1.132 (-0.667, 2.931) | 0.184 | 0.306 (-1.227, 1.838) | 0.696 | 0.449 (-1.057, 1.955) | 0.559 |
| FEV1/FVC ratio | -0.003 (-0.01, 0.003) | 0.302 | -0.002 (-0.008, 0.005) | 0.627 | -0.002 (-0.008, 0.005) | 0.617 |
| PEF, litres/min | 1.623 (-7.634, 10.88) | 0.307 | -2.691 (-11.577, 6.194) | 0.553 | -1.995 (-10.722, 6.732) | 0.654 |

We estimated the link between rationing expose and lung function measurements using ordinary least squares. Model 1 adjusted for age and sex. Model 2 additionally adjusted for race (white vs not), household income, Townsend deprivation index, smoking status, alcohol consumption, personal medical condition (cardiovascular disease, hypertension, diabetes, digestive disease, kidney disease and liver disease), parental lung disease, maternal smoking around birth, and whether breastfed as a baby. Model 3 adjusted for terms in Model 2 and calendar month of birth and survey year. FEV1 = forced expiratory volume in 1 second; FVC = forced vital capacity; PEF = peak expiratory flow.

**Supplementary Table 15.** Peak expiratory flow in the contemporaneous control group (HRS): participants born between October 1951 and June 1954 versus participants born between July 1954 and March 1956.

|  | Coefficient | 95%CI Lower limit | 95% CI Upper limit | P value |
| --- | --- | --- | --- | --- |
| Model 1 | 2.853 | -5.836 | 11.542 | 0.520 |
| Model 2 | -0.269 | -10.778 | 10.239 | 0.960 |
| Model 3 | 0.562 | -9.901 | 11.024 | 0.916 |

We estimated the link between rationing expose and peak expiratory flow using ordinary least squares. Model 1 adjusts for age, sex, and race. Model 2 adjusts for model 1 plus education, marital status, total wealth, smoking status, drinking status, and physical activity. Model 3 adjusts for model 2 plus hypertension, diabetes, and cardiovascular disease.

**Supplementary Table 16.** Spirometry in Rationed and Non-Rationed Participants of the ELSA Cohort.

| Spirometry | Coefficient | 95%CI Lower limit | 95% CI Upper limit | P value |
| --- | --- | --- | --- | --- |
| FVC, liter |  |  |  |  |
| Model 1 | 0.124 | 0.008 | 0.240 | 0.036 |
| Model 2 | 0.131 | 0.016 | 0.247 | 0.026 |
| Model 3 | 0.131 | 0.015 | 0.246 | 0.026 |
| FEV1, liter |  |  |  |  |
| Model 1 | 0.004 | -0.075 | 0.083 | 0.922 |
| Model 2 | 0.002 | -0.078 | 0.081 | 0.969 |
| Model 3 | 0.001 | -0.079 | 0.080 | 0.983 |
| PEF, liter per minute |  |  |  |  |
| Model 1 | 0.263 | -14.156 | 14.682 | 0.971 |
| Model 2 | -0.099 | -14.441 | 14.243 | 0.989 |
| Model 3 | -0.091 | -14.418 | 14.236 | 0.990 |

We estimated the link between rationing expose and lung function indices using ordinary least squares. Model 1 adjusts for age, sex, and race. Model 2 adjusts for model 1 plus education, marital status, family wealth, smoking status, drinking status, and physical activity. Model 3 adjusts for model 2 plus hypertension, diabetes, and cardiovascular disease. FEV1 = forced expiratory volume in 1 second; FVC = forced vital capacity; PEF = peak expiratory flow.


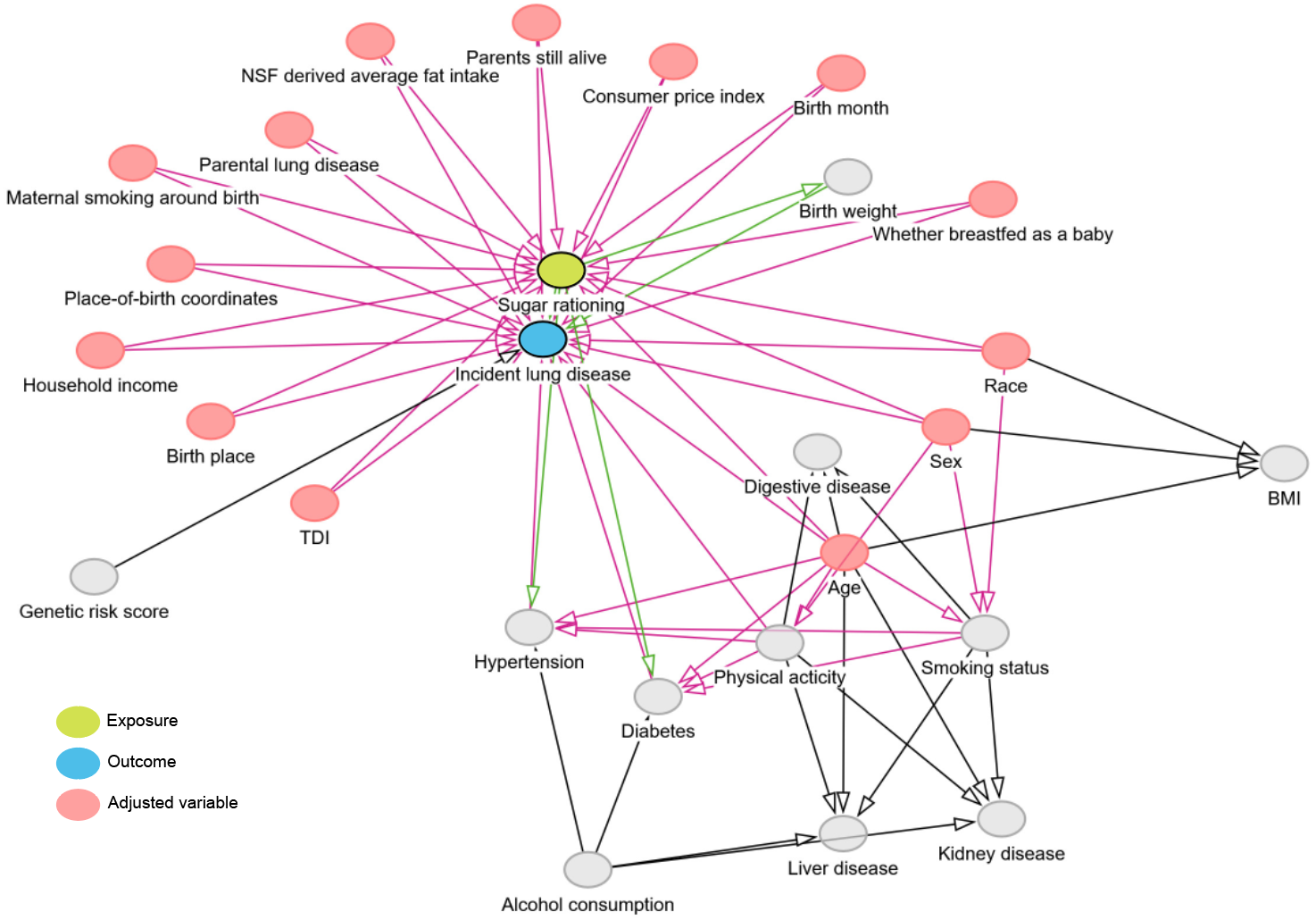


**Supplementary Figure 1.** Directed Acyclic Graph. The directed acyclic graph above was generated via the R package 'dagitty'.^40^ NSF: National Food Survey; TDI = Townsend deprivation index; BMI = Body Mass Index.

**
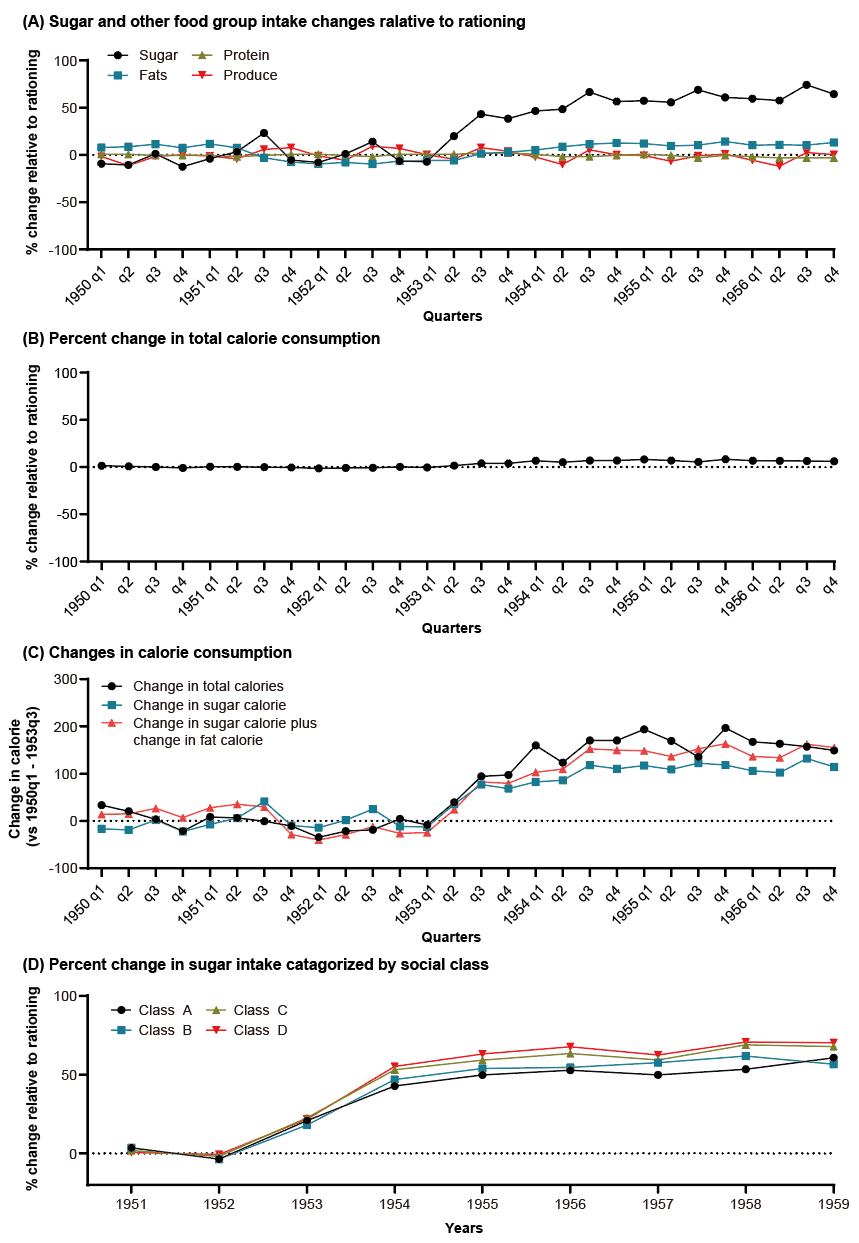
****Supplementary Figure 2.** Annual variation of food and food-related variables during the study period. For detailed information on the data used in the figure, see Supplementary Material 2 or download historical NSF data from <https://www.gov.uk/government/statistics/family-food-historic-reports>. Panels A, B, and C use the average consumption between 1950 Q1 and 1953 Q3 as the reference value. Panel D uses the average of 1951 and 1952 as the reference. Panels A, B, and D show percentage changes. Panel C shows raw changes.

**
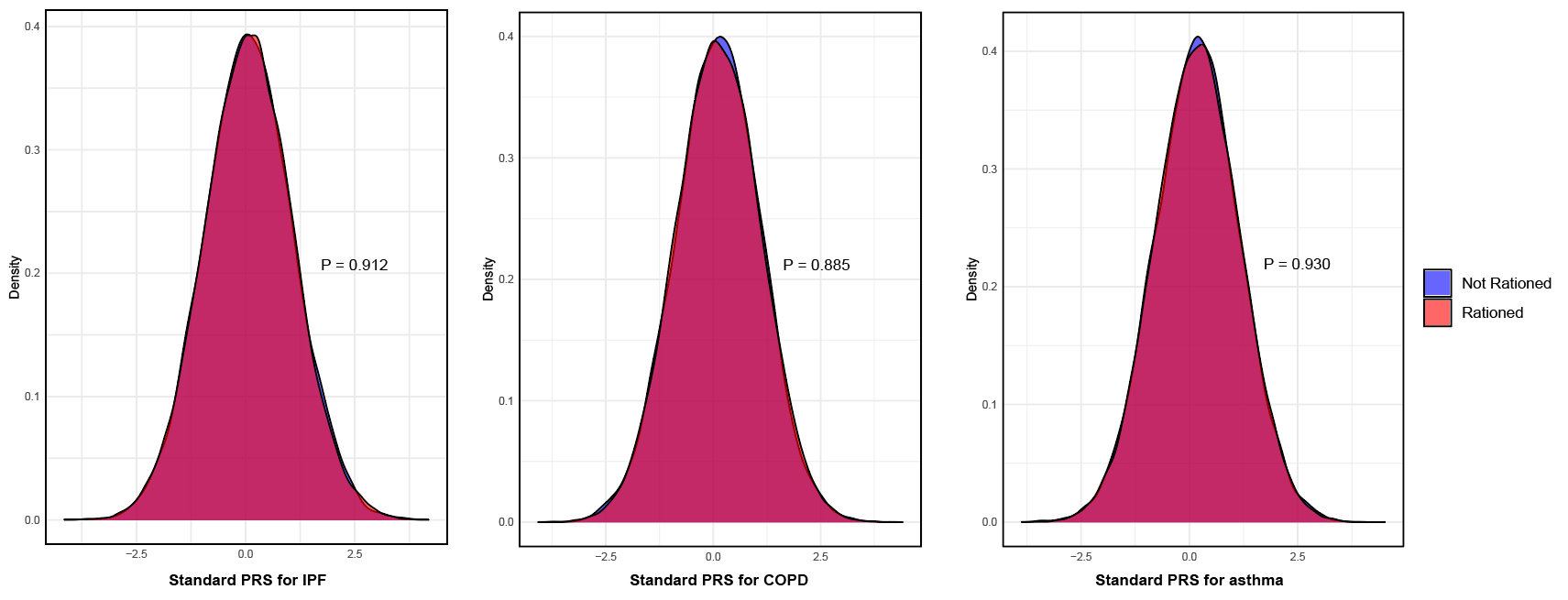
****Supplementary Figure 3.** Comparative density distributions of PRS (IPF, COPD and asthma) between rationed and not rationed groups.

The difference between the two groups' PRS was assessed using a t-test. IPF = idiopathic pulmonary fibrosis; COPD = chronic obstructive pulmonary disease.


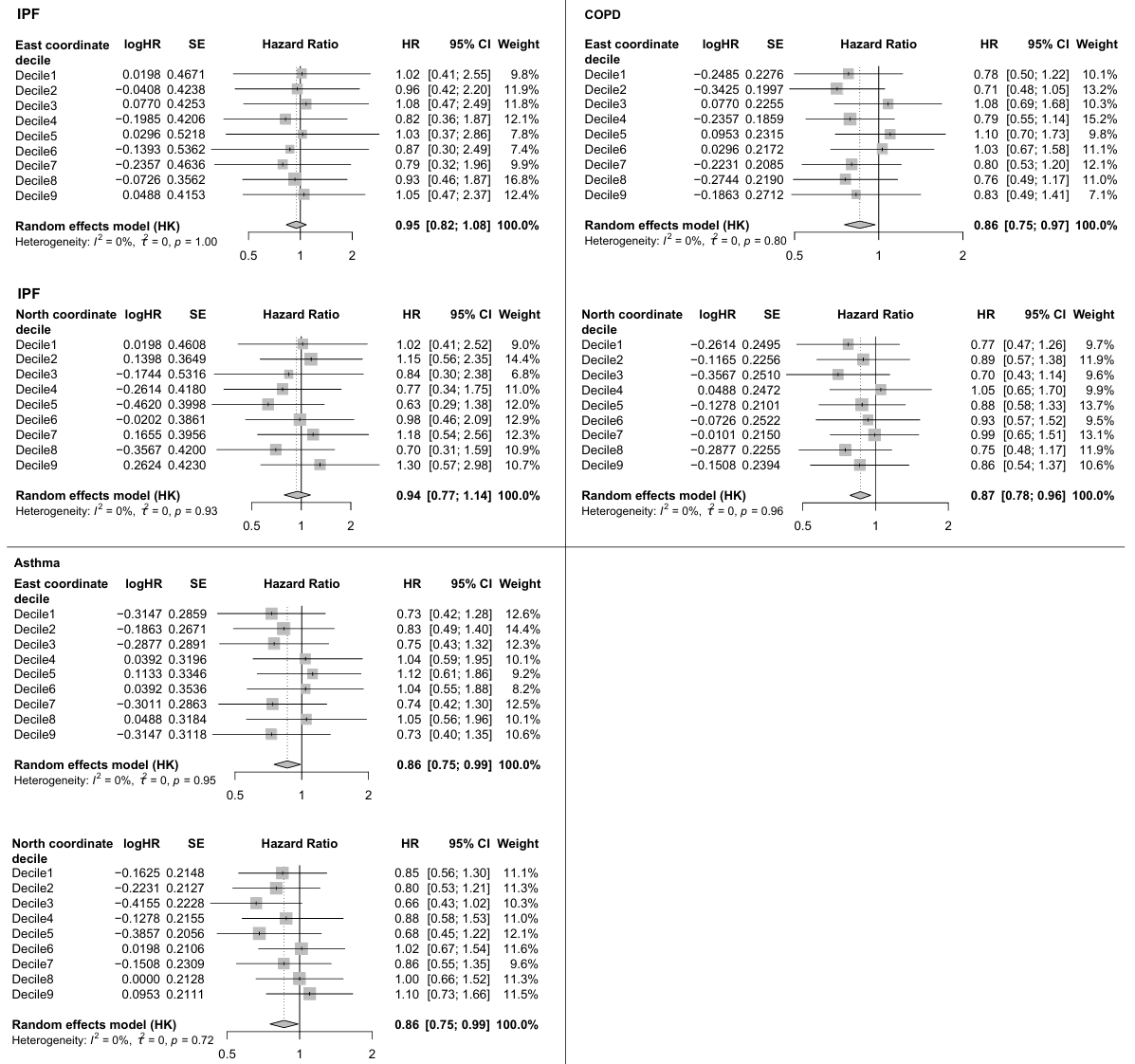


**Supplementary Figure 4.** Meta-analysis of hazard ratios for sugar rationing and respiratory outcomes across deciles of east and north coordinates.

Forest plots display HRs and 95% CIs for the association between early-life sugar rationing exposure and risk of IPF, COPD, and asthma, stratified by deciles of east and north coordinates at birth. Meta-analyses were performed using a random-effects model (Hartung-Knapp method). The pooled HR and corresponding 95% CI for each outcome are shown at the bottom of each plot. Measures of heterogeneity included I², τ², and Q-test p-value. All models were adjusted for age, sex, race, calendar month of birth, and survey year. IPF = idiopathic pulmonary fibrosis; COPD = chronic obstructive pulmonary disease; HR = hazard ratio; CI = confidence interval


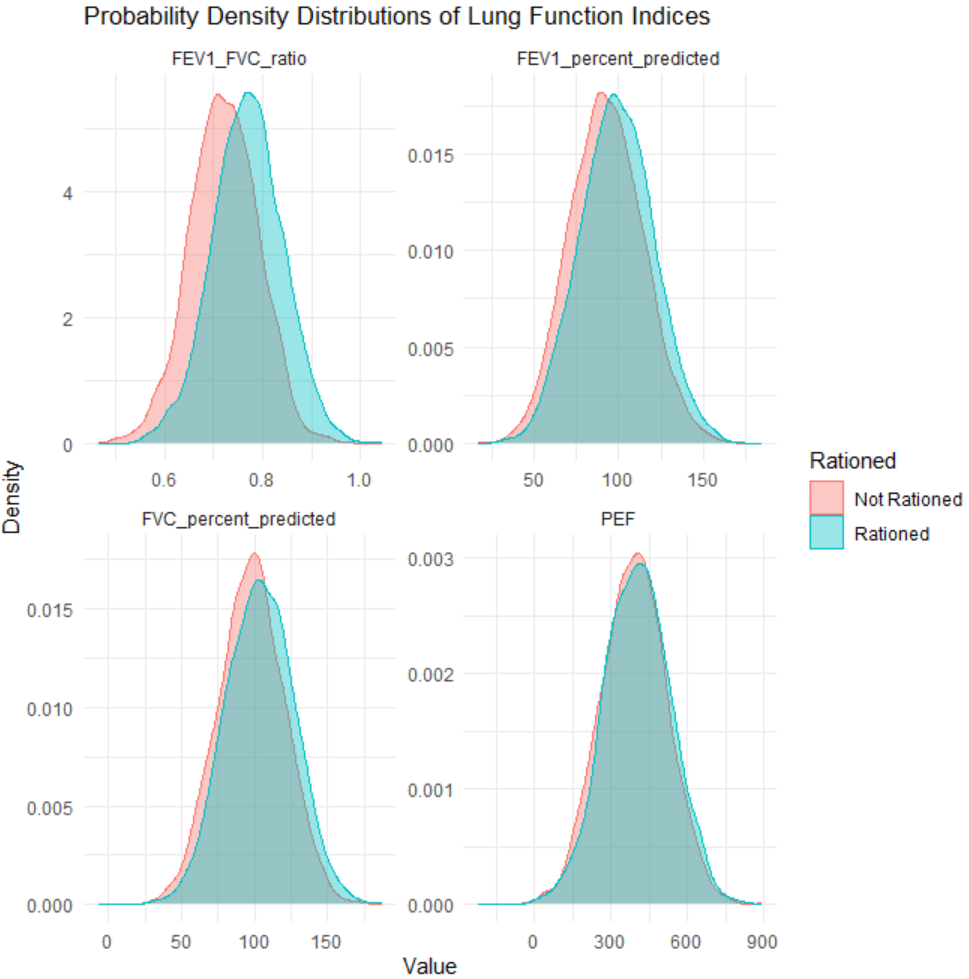
**Supplementary Figure 5.** Comparative density distributions of lung function metrics (FEV1% predicted, FVC% predicted, FEV1/FVC ratio and PEF) between rationed and not rationed groups.

A total of 53,581 participants with available spirometry data was analyzed. FEV1 = forced expiratory volume in 1 second; FVC = forced vital capacity; PEF = peak expiratory flow.

**
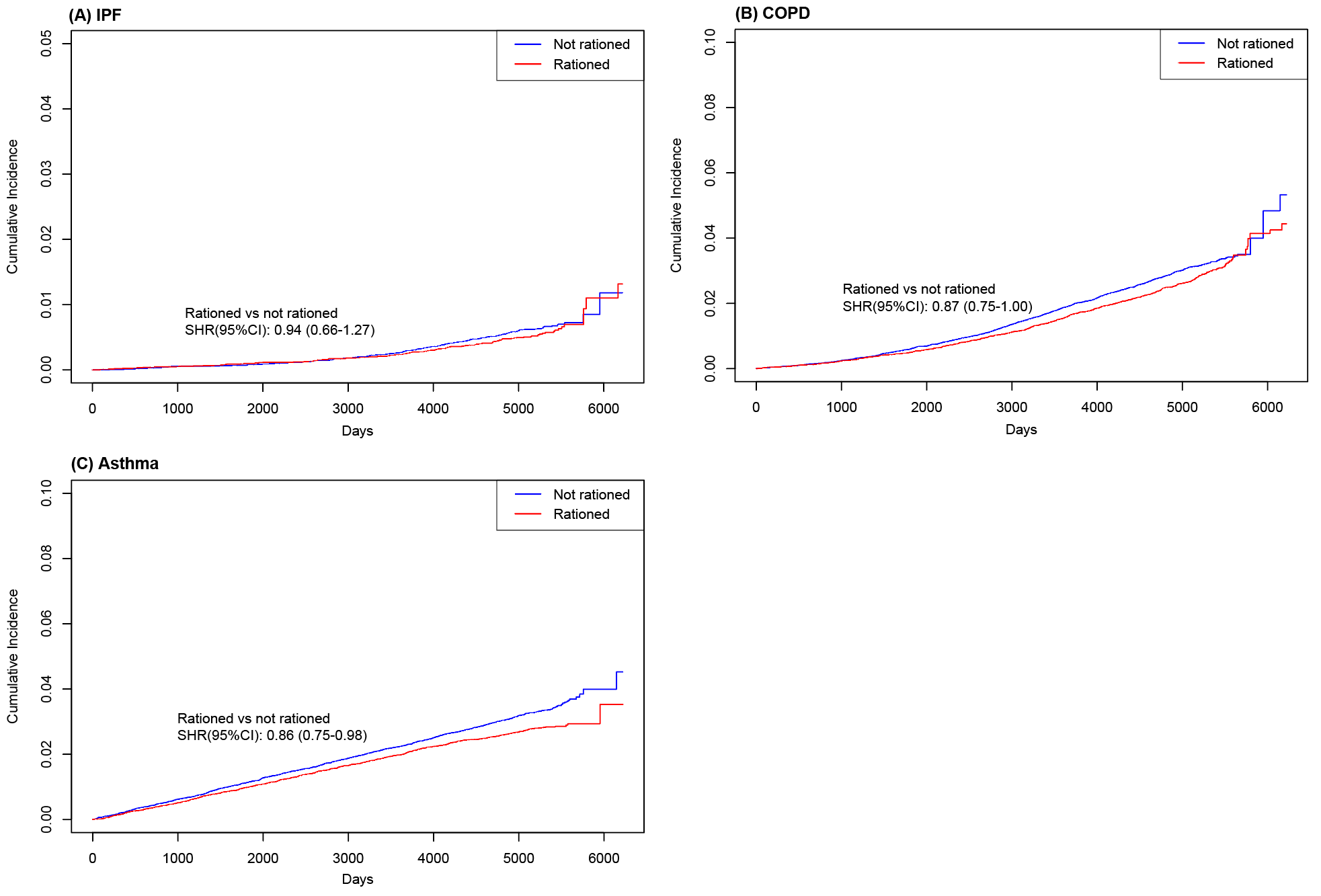
****Supplementary Figure 6.** Cumulative incidence of chronic respiratory disease between individuals exposed to sugar rationing and never-rationed adults.

A Fine and Gray model was used to adjust for competing risks, with the competing events being non-respiratory mortality. There were 3164 competing risk events recorded for IPF, 2879 for COPD, and 3166 for asthma. IPF = idiopathic pulmonary fibrosis; COPD = chronic obstructive pulmonary disease. CI = confidential interval; SHR, sub-distribution hazard ratio.


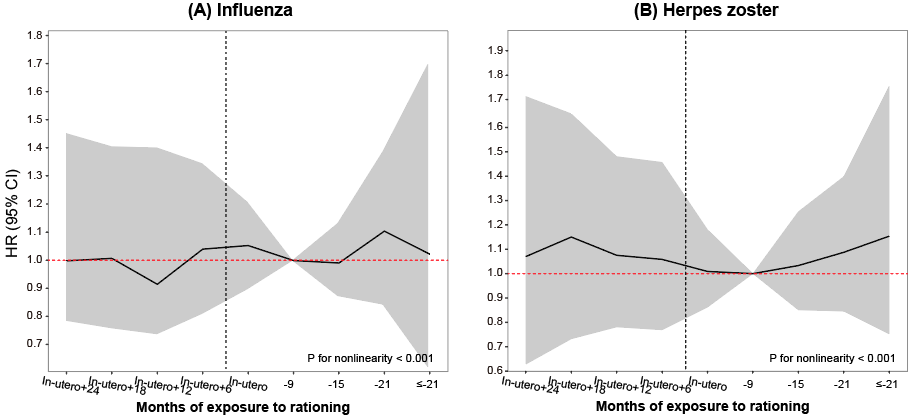
**Supplementary Figure 7.** Hazard ratios for placebo outcomes (influenza [n=208] and herpes zoster [n=154]) by various levels of rationing exposure.

Parametric hazard models based on the Gompertz distribution were used. Penalized splines were adjusted for age, sex. race, birth location, calendar month of birth, household income, Townsend deprivation index, real food prices (adjusted for the consumer price index), parental lung disease, whether parents still alive, maternal smoking around birth, whether breastfed as a baby, the social average fat intake around the period of birth (derived from the NSF), baseline lifestyle factors (smoking status, alcohol intake and physical activity), baseline medical conditions (CVD, hypertension, diabetes, digestive disease, kidney disease, and liver disease) and survey year. The shaded area represents the 95% confidence interval. The vertical dashed black line marks the end of sugar rationing.


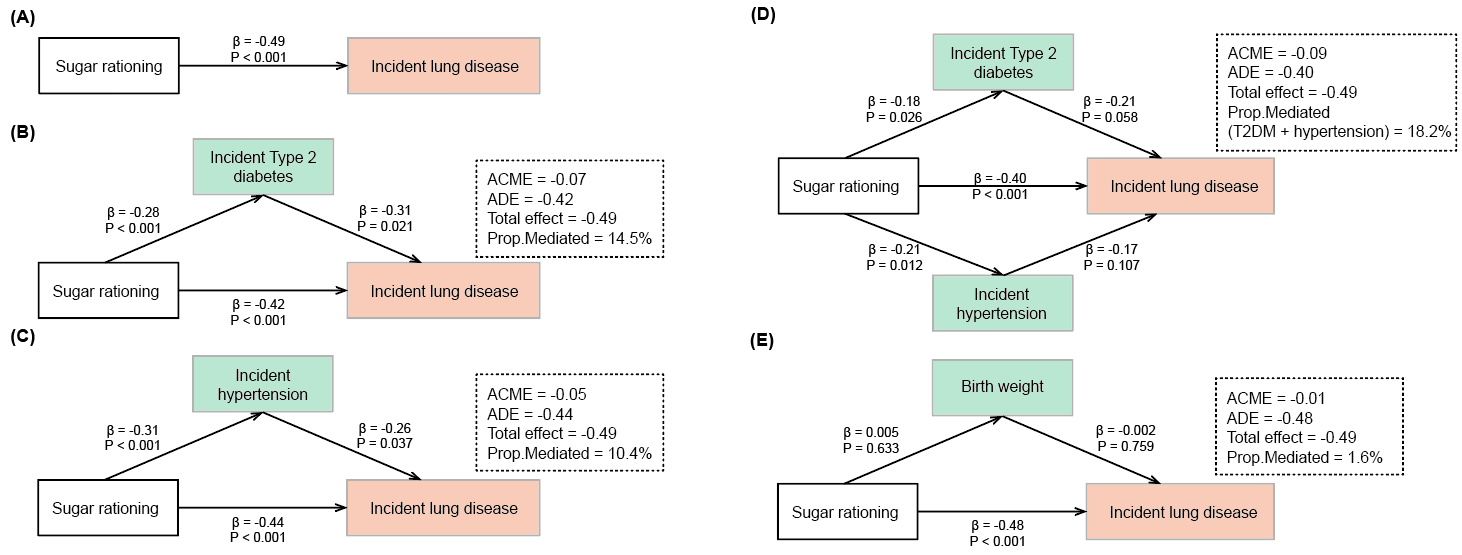
**Supplementary Figure 8.** The standard mediation analysis investigating the proportion mediated by diabetes, hypertension, and birth weight in the relationship between sugar rationing and lung disease. Mediation analysis was conducted using structural equation modeling (SEM) with the lavaan R package. ^35^ Covariates in Model 2 were adjusted for in the linear regression model. CVD = cardiovascular disease; ACME = average causal mediated effect; ADE = average direct effect; T2DM = type 2 diabetes mellitus; Prop. Mediated = proportion mediated.

**
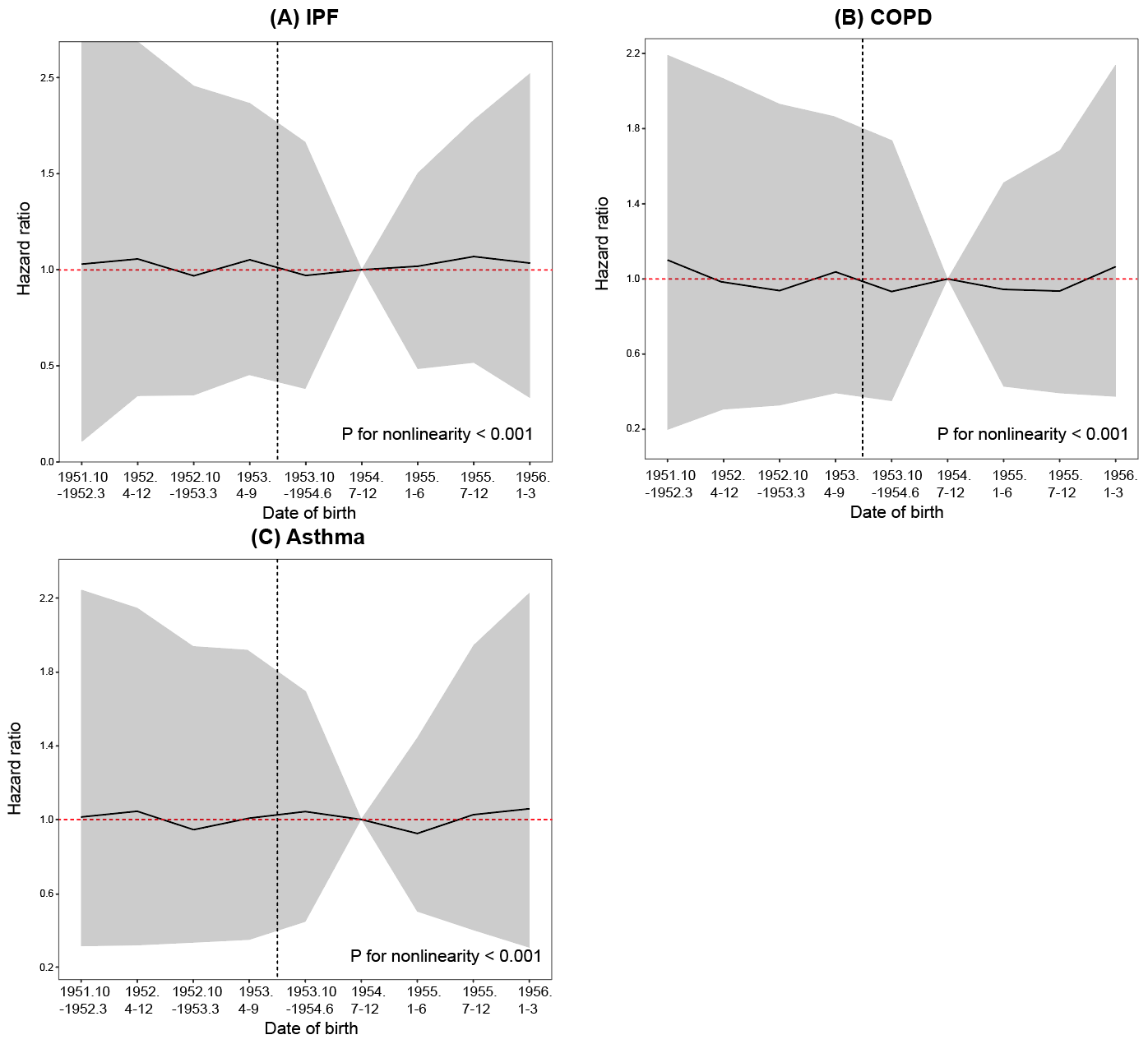
****Supplementary Figure 9.** Hazard ratios for different chronic respiratory diseases by date of birth in individuals born outside of UK and have not experienced sugar rationing (n=5,804).

Parametric hazard models based on the Gompertz distribution were applied. Penalized splines were adjusted for sex, race (white vs. non-white), household income, Townsend deprivation index, smoking status, alcohol consumption, personal medical conditions (cardiovascular disease, hypertension, diabetes, digestive diseases, kidney disease, and liver disease), parental lung disease, maternal smoking around birth, breastfeeding history, birth month and survey year. The shaded area represents the 95% confidence interval. The vertical dashed black line marks the end of sugar rationing. IPF = idiopathic pulmonary fibrosis; COPD = chronic obstructive pulmonary disease.

**Reference:**

1. The 2030 Agenda for Sustainable Development--United Nations, Department of Economic and Social Affairs. <https://sdgs.un.org/2030agenda>.

2. Grummon, A. H.; Lockwood, B. B.; Taubinsky, D.; Allcott, H., Designing better sugary drink taxes. *Science* **2019,** *365* (6457), 989-990.

3. J. Buxton, E. Tipping, S. Rozee, E. Morgan, “Mortality in England and Wales: past and projected trends in average lifespan” (Office for National Statistics, 2022); <https://www.ons.gov.uk/peoplepopulationandcommunity/birthsdeathsandmarriages/lifeexpectancies/articles/mortalityinenglandandwales/pastandprojectedtrendsinaveragelifespan>.

4. Herrick, K. A.; Fryar, C. D.; Hamner, H. C.; Park, S.; Ogden, C. L., Added sugars intake among US infants and toddlers. *Journal of the Academy of Nutrition and Dietetics* **2020,** *120* (1), 23-32.

5. Cioffi, C. E.; Figueroa, J.; Welsh, J. A., Added sugar intake among pregnant women in the United States: National Health and nutrition examination survey 2003-2012. *Journal of the Academy of Nutrition and Dietetics* **2018,** *118* (5), 886-895. e1.

6. American Heart Association. “Added Sugars.” <https://www.heart.org/en/healthy-living/healthy-eating/eat-smart/sugar/added-sugars>.

7. Snetselaar, L. G.; de Jesus, J. M.; DeSilva, D. M.; Stoody, E. E., Dietary guidelines for Americans, 2020–2025: understanding the scientific process, guidelines, and key recommendations. *Nutrition today* **2021,** *56* (6), 287-295.

8. Zweiniger-Bargielowska, I., *Austerity in Britain: rationing, controls, and consumption, 1939-1955*. OUP Oxford: 2000.

9. Prynne, C.; Paul, A.; Price, G.; Day, K.; Hilder, W.; Wadsworth, M., Food and nutrient intake of a national sample of 4-year-old children in 1950: comparison with the 1990s. *Public Health Nutr.* **1999,** *2* (4), 537-547.

10. Hollingsworth, D. F., Rationing and economic constraints on food consumption in Britain since the second world war. **1983**.

11. Miyake, K.; Kushima, M.; Shinohara, R.; Horiuchi, S.; Otawa, S.; Akiyama, Y., et al., Maternal smoking status before and during pregnancy and bronchial asthma at 3 years of age: a prospective cohort study. *Scientific Reports* **2023,** *13* (1), 3234.

12. Wada, T.; Adachi, Y.; Murakami, S.; Ito, Y.; Itazawa, T.; Tsuchida, A., et al., Maternal exposure to smoking and wheezing phenotypes in children: a cohort study of the Japan Environment and Children’s Study. *BMC Pediatr.* **2024,** *24* (1), 624.

13. He, H.; He, M.-M.; Wang, H.; Qiu, W.; Liu, L.; Long, L., et al., In utero and childhood/adolescence exposure to tobacco smoke, genetic risk, and lung cancer incidence and mortality in adulthood. *Am. J. Respir. Crit. Care Med.* **2023,** *207* (2), 173-182.

14. McEvoy, C. T.; Spindel, E. R., Pulmonary effects of maternal smoking on the fetus and child: effects on lung development, respiratory morbidities, and life long lung health. *Paediatr. Respir. Rev.* **2017,** *21*, 27-33.

15. Gracner, T.; Boone, C.; Gertler, P. J., Exposure to sugar rationing in the first 1000 days of life protected against chronic disease. *Science* **2024,** *386* (6725), 1043-1048.

16. Jayaratne, S.; Prestipino, L.; Polson, J. W., Maternal High Fat‐High Sucrose Diet is Associated with Elevated Offspring Hypothalamic Oxidative Stress and Blood Pressure Responses to Psychological Stress. *The FASEB Journal* **2017,** *31*, 852.7-852.7.

17. He, S.; Stein, A. D., Early-life nutrition interventions and associated long-term cardiometabolic outcomes: a systematic review and meta-analysis of randomized controlled trials. *Advances in Nutrition* **2021,** *12* (2), 461-489.

18. Zhou, L.-Y.; Deng, M.-Q.; Zhang, Q.; Xiao, X.-H., Early-life nutrition and metabolic disorders in later life: A new perspective on energy metabolism. *Chin. Med. J. (Engl.)* **2020,** *133* (16), 1961-1970.

19. Olivier-Van Stichelen, S.; Rother, K. I.; Hanover, J. A., Maternal exposure to non-nutritive sweeteners impacts progeny’s metabolism and microbiome. *Frontiers in microbiology* **2019,** *10*, 1360.

20. Crapo, R. O.; Hankinson, J. L.; Irvin, C.; MacIntyre, N. R.; Voter, K.; Wise, R., et al., Standardization of spirometry: 1994 update. *Am. J. Respir. Crit. Care Med.* **1995,** *152* (3), 1107-1136.

21. Staff, H., HRS core interview sample sizes and response rates. *Ann Arbor, MI: Survey Research Center, Institute for Social Research, University of Michigan* **2023**.

22. Sonnega, A.; Faul, J. D.; Ofstedal, M. B.; Langa, K. M.; Phillips, J. W.; Weir, D. R., Cohort profile: the health and retirement study (HRS). *Int. J. Epidemiol.* **2014,** *43* (2), 576-585.

23. Barker, D. J., The developmental origins of adult disease. *J. Am. Coll. Nutr.* **2004,** *23* (sup6), 588S-595S.

24. Stocks, J.; Hislop, A.; Sonnappa, S., Early lung development: lifelong effect on respiratory health and disease. *The lancet Respiratory medicine* **2013,** *1* (9), 728-742.

25. Agosti, M.; Tandoi, F.; Morlacchi, L.; Bossi, A., Nutritional and metabolic programming during the first thousand days of life. *La Pediatria Medica e Chirurgica* **2017,** *39* (2).

26. Howie, B. N.; Donnelly, P.; Marchini, J., A flexible and accurate genotype imputation method for the next generation of genome-wide association studies. *PLoS Genet.* **2009,** *5* (6), e1000529.

27. Loh, P.-R.; Tucker, G.; Bulik-Sullivan, B. K.; Vilhjálmsson, B. J.; Finucane, H. K.; Salem, R. M., et al., Efficient Bayesian mixed-model analysis increases association power in large cohorts. *Nat. Genet.* **2015,** *47* (3), 284-290.

28. Purcell, S.; Neale, B.; Todd-Brown, K.; Thomas, L.; Ferreira, M. A.; Bender, D., et al., PLINK: a tool set for whole-genome association and population-based linkage analyses. *The American journal of human genetics* **2007,** *81* (3), 559-575.

29. Allen, R. J.; Stockwell, A.; Oldham, J. M.; Guillen-Guio, B.; Schwartz, D. A.; Maher, T. M., et al., Genome-wide association study across five cohorts identifies five novel loci associated with idiopathic pulmonary fibrosis. *Thorax* **2022,** *77* (8), 829-833.

30. Sakornsakolpat, P.; Prokopenko, D.; Lamontagne, M.; Reeve, N. F.; Guyatt, A. L.; Jackson, V. E., et al., Genetic landscape of chronic obstructive pulmonary disease identifies heterogeneous cell-type and phenotype associations. *Nat. Genet.* **2019,** *51* (3), 494-505.

31. Zhu, Z.; Zhu, X.; Liu, C.-L.; Shi, H.; Shen, S.; Yang, Y., et al., Shared genetics of asthma and mental health disorders: a large-scale genome-wide cross-trait analysis. *Eur. Respir. J.* **2019,** *54* (6).

32. Ripatti, S.; Tikkanen, E.; Orho-Melander, M.; Havulinna, A. S.; Silander, K.; Sharma, A., et al., A multilocus genetic risk score for coronary heart disease: case-control and prospective cohort analyses. *The Lancet* **2010,** *376* (9750), 1393-1400.

33. Choi, S. W.; Mak, T. S.-H.; O’Reilly, P. F., Tutorial: a guide to performing polygenic risk score analyses. *Nat. Protoc.* **2020,** *15* (9), 2759-2772.

34. Inouye, M.; Abraham, G.; Nelson, C. P.; Wood, A. M.; Sweeting, M. J.; Dudbridge, F., et al., Genomic risk prediction of coronary artery disease in 480,000 adults: implications for primary prevention. *J. Am. Coll. Cardiol.* **2018,** *72* (16), 1883-1893.

35. Rosseel, Y., lavaan: An R package for structural equation modeling. *Journal of statistical software* **2012,** *48* (1), 1-36.

36. Fairchild, A. J.; McDaniel, H. L., Best (but oft-forgotten) practices: Mediation analysis1, 2. *The American journal of clinical nutrition* **2017,** *105* (6), 1259-1271.

37. Hu, L. t.; Bentler, P. M., Cutoff criteria for fit indexes in covariance structure analysis: Conventional criteria versus new alternatives. *Structural equation modeling: a multidisciplinary journal* **1999,** *6* (1), 1-55.

38. Tierney, N. J., Cook, D. H., McBain, M., & Fay, C. (2023). naniar: Data Structures, Summaries, and Visualisations for Missing Data. R package version 1.0.0. <https://CRAN.R-project.org/package=naniar>.

39. Lam, J. C.; Han, Y.; Wang, S.; Li, V. O.; Pollitt, M.; Warde, P., A comparative study of air pollution trends in historical London and contemporary Beijing. In *In search of good energy policy*, Cambridge University Press: 2019; pp 282-304.

40. Textor, J.; Van der Zander, B.; Gilthorpe, M. S.; Liśkiewicz, M.; Ellison, G. T., Robust causal inference using directed acyclic graphs: the R package ‘dagitty’. *Int. J. Epidemiol.* **2016,** *45* (6), 1887-1894.

**Supplementary material 2**

Sugar Restriction in the First 1000 Days after Conception and Long-Term Respiratory Health: A Quasi-experiment Study

Jiazhen Zheng et al.

Detailed annual data on food intake, Food Price Index, CPI, and related socioeconomic indicators are available in “Supplementary Material 2.zip” (downloadable via https://drive.google.com/file/d/1rNm4056IxcKJl_lLYQYhagdMyx6GbKsn/view?usp=sharing). Alternatively, these data can be accessed from the Family Food historic reports via https://www.gov.uk/government/statistics/family-food-historic-reports.
